# Electroacupuncture for acute gouty arthritis: a systematic review and meta-analysis of randomized controlled trials

**DOI:** 10.3389/fimmu.2023.1295154

**Published:** 2024-01-04

**Authors:** Zhichao Ni, Qinwen Xiao, Zihao Xia, Kunlin Kuang, Bingzun Yin, Dezhong Peng

**Affiliations:** ^1^ College of Acupuncture-Moxibustion and Tuina, Chengdu University of Traditional Chinese Medicine, Chengdu, China; ^2^ West China Second University Hospital, Sichuan University, Chengdu, China

**Keywords:** electroacupuncture, acute gouty arthritis, gout, systematic review, meta-analysis

## Abstract

Acute gouty arthritis (AGA) is a metabolic disorder in which recurrent pain episodes can severely affect the quality of life of gout sufferers. Electroacupuncture (EA) is a non-pharmacologic therapy. This systematic review aimed to assess the efficacy and safety of electroacupuncture in treating acute gouty arthritis. We searched eight Chinese and English databases from inception to July 30, 2023, and 242 studies were retrieved. Finally, 15 randomized controlled trials (n=1076) were included in a meta-analysis using Review Manager V.5.4.1. meta-analysis results included efficacy rate, visual rating scale (VAS) for pain, serum uric acid level (SUA), immediate analgesic effect, and incidence of adverse events. Electroacupuncture (or combined non-pharmacologic) treatment of AGA was significantly different from treatment with conventional medications (RR = 1.14, 95% confidence interval CI = 1.10 to 1.19, P < 0.00001). The analgesic effect of the electroacupuncture group was superior to that of conventional Western drug treatment (MD = -2.26, 95% CI = -2.71 to -1.81, P < 0.00001). The electroacupuncture group was better at lowering serum uric acid than the conventional western drug group (MD =-31.60, CI -44.24 to -18.96], P < 0.00001). In addition, electroacupuncture combined with Western drugs had better immediate analgesic effects than conventional Western drug treatment (MD = -1.85, CI -2.65 to -1.05, P < 0.00001). Five studies reported adverse events in the electroacupuncture group versus the drug group, including 19 cases of gastrointestinal symptoms and 6 cases of neurological symptoms (RR = 0.20, 95% CI = 0.04 to 0.88, P = 0.03).

**Systematic review registration:**

https://www.crd.york.ac.uk/prospero/display_record.php?RecordID=450037, identifier CRD42023450037.

## Introduction

Acute gouty arthritis (AGA) is the typical first symptom in patients with gout. It presents as acute inflammatory arthritis with periodic episodes of severe pain, often involving the joints of the lower extremities (1). The pain experienced during acute inflammatory attacks in patients with gout is often described as excruciating stabbing, biting, burning, or throbbing sensations (2). Furthermore, joint swelling and warmth can accompany these pain episodes in varying degrees (1, 3). As a result of these intermittent periods of pain, the affected area is significantly limited in its functionality, making it difficult to walk and causing a fear of even minimal physical contact (4). While acute gout attacks primarily affect the lower extremities, they can also occur in the elbow, wrist, and hand joints in patients with chronic, poorly controlled disease (2, 5). Additionally, the recurrence of gout is unpredictable, and the chances of experiencing repeated flare-ups are influenced by the severity of hyperuricemia (6). AGA attacks cause immense, intolerable pain to the patient and, due to their unpredictability, severely affect activities of daily living, mood, and social functioning (7, 8). The distress caused by the pain to the patient was found to be immense, especially the severe pain during severe flare-ups, which almost completely limits the possibility of walking and standing and can cause more distress in the knee than in other small joints. A study that included 210 patients with gout flare criteria reported that some patients considered their symptoms to be gout flare but were not recognized by specialists who followed the diagnostic criteria, suggesting that the distress caused by acute gout attacks may be underestimated (3).

Epidemiologic investigations have shown that the prevalence of gout varies depending on the population studied, ranging from 1% to 6.8%, being higher in men than in women and that its prevalence and incidence are increasing globally. Notably, the highest prevalence is found in marine countries, particularly indigenous and South Pacific island populations, while developing countries have the lowest prevalence (9). It is believed that elevated serum urate levels (hyperuricemia) lead to the deposition of monosodium urate crystals in joints, tendons, and other tissues, causing recurrent episodes of AGA (10). Furthermore, factors contributing to this include metabolic syndrome, chronic kidney disease, and the use of medications like diuretics and cyclosporine. Environmental exposures, such as consuming purine-rich foods like red meat and seafood, drinking alcohol (especially purine-rich beer), and consuming sugar-sweetened beverages, also raise serum urate concentrations and the risk of gout (11). There is evidence of a high comorbidity burden at the time of gout diagnosis and a higher risk of new comorbidities in patients with first-episode gout compared to the general population (12).

The pathogenesis of AGA is an acute inflammatory response induced by sodium urate crystals. The NLRP3 inflammatory vesicles rely on a dual signaling system initiated by TLR4 and TLR2 and stimulated by urate crystals (2, 13, 14). Certain factors like free fatty acids, induced by consuming a large meal or alcohol, as well as the intestinal microbiota and other microbial constituents, can stimulate the initial signal, leading to an acute inflammatory response caused by the sodium urate crystals (15). These crystals then serve as a second activation signal, initiating a signaling cascade that recruits neutrophils and other cells to the site where the crystals are deposited, thereby driving the local acute immune response.

In current protocols for controlling and managing acute gout attacks, one or more standard oral anti-inflammatory treatments for gout are used (i.e., nonsteroidal anti-inflammatory drugs, colchicine, corticosteroids). However, these medications usually require at least 12-24 hours to achieve robustness and may even take several days to a week to fully effect (16). In addition, these oral medications have different contraindications and adverse effects (17). So, safer and more effective treatments are still needed.

Therefore, it is of clinical importance to find alternative methods with minimal toxic side effects to reduce gouty arthritis pain. One potential treatment that meets these criteria is acupuncture. Acupuncture is a traditional Chinese medicine (TCM) treatment that is part of complementary and alternative medicine (CAM) in modern medicine. Acupuncture meets the necessary criteria to be considered a potential tool, including safety and a certain level of effectiveness. It enjoys significant popularity among patients, particularly those suffering from pain-related conditions (18). Animal studies investigating inflammatory pain have demonstrated that electroacupuncture engages numerous bioactive substances to alleviate pain, leading to its ultimate suppression through peripheral and central mechanisms (19). A 2023 animal study found that electroacupuncture reduced ros-mediated hyperactivation of ankle NLRP3 inflammatory vesicles and upregulation of sensory neuron TRPV1 may contribute to the interventional effects of EA in animal models of gouty arthritis (20).

Although there are no guidelines, electroacupuncture has long been used to treat patients with AGA (21–23), yet there is no meta-analysis on electroacupuncture for AGA. (Elaboration purpose) Therefore, this systematic review aims to assess the safety and effectiveness of electroacupuncture for treating acute gouty arthritis, aiming to offer practical guidance in clinical practice.

## Materials and methods

We are pre-registered with the review protocol (https://www.crd.york.ac.uk/prospero/), registration number CRD42023450037. All reviewers undergo the same training to ensure completeness and consistency throughout the review process. We developed methods based on the Preferred Reporting Items for Systematic Reviews and Meta-Analyses (PRISMA) standards and meta-analysis protocols, as well as the Cochrane Handbook for Systematic Reviews of Interventions, to ensure the accuracy of these systematic evaluations and meta-analyses (24).

### Diagnostic criteria

Patients included in the study had to be diagnosed with gouty arthritis according to established diagnostic criteria as follows: 2015 Gout classification criteria: an American College of Rheumatology/European League Against Rheumatism collaborative initiative (25). AGA patients did not include joint inflammation due to other diseases.

### Inclusion and exclusion criteria

Studies that fulfilled the given criteria were considered suitable for inclusion:1) the study was conducted in individuals with a confirmed diagnosis of AGA; 2) the study design was an RCT; 3) Electroacupuncture was the main therapy, either alone or in conjunction with other approaches.; and 4) The language was restricted to only English or Chinese. Exclusion criteria were: 1) animal studies, case reports, self-controlled, non-randomized controlled trials, and 2) repeated published studies.

### Search strategy

We searched eight databases electronically and manually for all electroacupuncture randomized controlled trials of acute gouty arthritis from the beginning of the database until July 31, 2023, by searching the databases. The databases included PubMed, Web of Science, Embase, Cochrane Library, China Knowledge Network (CNKI), China Biomedical Literature Database (CBM), Chinese Scientific Journal Database (VIP), and Wanfang. Medical subject headings (MeSH) and keywords were utilized to search for the articles. (e.g., acute gouty arthritis, electroacupuncture, and randomized controlled trials) combined with Boolean logic operators. The electronic search included the utilization of the subsequent search terms: (Gouty Arthritis [MeSH Terms] OR Gouty Arthritides [Title/Abstract] OR Acute Gouty Arthritis [Title/Abstract] OR Gouts [Title/Abstract]) AND (Electroacupuncture [MeSH Terms] OR Electroacupuncture therapy [Title/Abstract] OR Electroacupuncture treatment [Title/Abstract] OR Electroacupuncture [Title/Abstract]) AND (randomized controlled trial [Title/Abstract]). We adapted the search terms of different databases to suit their search criteria (**Supplementary Appendix**).

### Data collection and analysis

#### Study Selection

Two reviewers (ZCN and QWX) independently reviewed the screening results. The first review involved examining the title, abstract, and keywords. Afterward, a thorough examination of the full text of potential studies that matched the inclusion criteria was conducted. In the event of any disagreements that occur during this process, a resolution will be reached through consultation by a third reviewer. (DZP).

#### Data Extraction and Management

The following information will be obtained separately from the included RCTs by our researchers (KLK and BZY), independently using a pre-designed extraction scale: first author, date of publication, sample size, age, duration of the disease, intervention, acupoints, frequency, and duration of treatments, outcomes, efficacy, and adverse effects. Efforts will be made to reach out to the authors in case there is any missing or unclear information in the Randomized Control Trial (RCT). In case of any disagreements that occur during this process will be resolved either through consultation or by involving a third reviewer (DZP).

### Outcome measures

The main indicator is the effectiveness rate (26, 27). It is defined as the percentage of the total number of patients who are clinically cured (28, 29), who report that the treatment is significantly effective, and who report that the treatment is effective. It was measured based on two commonly used criteria. The total effective rate (%) calculated in this study = ([number of clinically cured + number of significantly effective + number of effective]/number of patients*100%). The different effective levels were defined as follows.

I) Clinical cure - symptoms and signs substantially improved, score ≥95%;

II) Apparently effective - symptoms and signs are significantly relieved, score decreases to 70% ~ 95%;

III) Effective - some relief of symptoms and signs, scores decreasing to 30% ~ 70%;

IV) Ineffective - no relief of symptoms and signs, score decreases to no more than 30%.

Secondary observations included pain rating scale (VAS), serum uric acid level, and occurrence of adverse events (e.g., gastrointestinal and neurological symptoms).

### Assessment of risk of bias

To assess the risk of bias, we will utilize the risk of bias tool in the Cochrane Handbook V.5.1.0. Each included study was independently assessed by two researchers (ZCN and ZHX). Based on the aforementioned tool, the risk of bias was eventually classified as low, high, or unclear. In case of any discrepancies, they were resolved through discussion involving the third author. (DZP).

### Measures of treatment effect

The analysis of the data was carried out using Review Manager version V.5.4.1. When there was no significant heterogeneity in the data, it was analyzed using a fixed effects model. Conversely, a random effects model was used in cases with significant group heterogeneity. Descriptive analysis methods were recommended in situations where quantitative analysis was not feasible. Effectiveness is a dichotomous variable, with only effectiveness and ineffectiveness as indicators. The Visual Assessment Scale (VAS) for pain was a manual entry. To enhance comparability and eliminate baseline error, we calculated the mean difference between the pre-treatment VAS values and the post-treatment VAS values in each study. The mean difference in VAS decreases after treatment compared to before was used to compare the statistical differences between the treatment and control groups. Serum uric acid levels in each study were obtained by testing in hospital laboratories, and the post-treatment test values of the test and control groups were directly selected as statistical parameters. The measurement for data on continuous variables was conducted using the mean difference (MD) with a 95% confidence interval [CI]. For data on dichotomous variables, the outcomes were measured using the risk ratio (RR) with a 95% CI.

### Level of evidence

The Grading of Recommendations, Assessment, Development, and Evaluation (GRADE) was used to evaluate the level of evidence for each outcome. The level of evidence was categorized as high, moderate, low, or very low. The evaluation of the level of evidence considered several domains, including risk of bias, imprecision, inconsistency, indirectness, publication bias, large magnitude of effect, dose-response, and confounding. The criteria set by the GRADE group were used to assess these domains.

### Assessment of heterogeneity and reporting bias

Heterogeneity was assessed using the Higgins test by calculating I^2^, and values of I^2^ greater than 50% could indicate significant heterogeneity. Potential reasons for the heterogeneity were examined through sensitivity and subgroup analyses as follows: 1) Different electro-acupuncture combinations, 2) Sample size, and 3) Frequency of electroacupuncture.

If there were more than 10 eligible studies, funnel plots were utilized to assess publication bias. At the same time, Egger’s and Begg’s tests were employed to verify the presence of publication bias.

## Results

### Study characteristics

**Figure 1:**
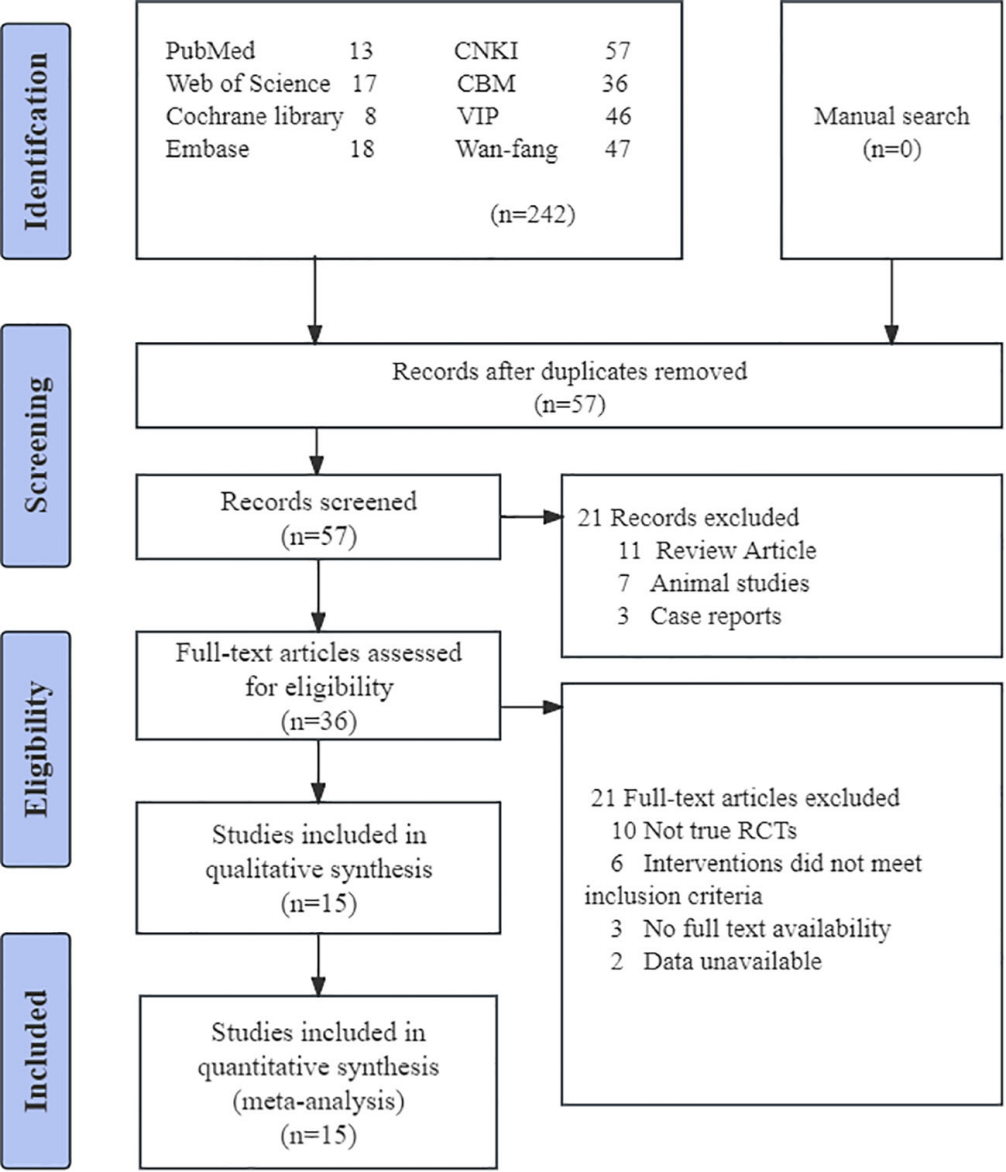
Flowchart of PRISMA. CNKI, China National Knowledge Infrastructure; CBM, China Biomedical Literature Database; VIP = China Science Journal Database.

Initially, 289 studies were included, and no extra articles were identified through a manual search. Following the assessment by two independent reviewers (ZCN and QWX), 15 RCTs were finally included in the study (21–23, 26, 27, 30–39), totaling 1076 subjects. **Figure 1** displays the study selection procedure. All 15 trials analyzed were single-center RCTs conducted in China, published in English and Chinese within the timeframe of 2005-2023. One of the studies was a master’s thesis in 2018 (26). In the 15 trials, 1076 patients with AGA, ranging in age from 20 to 72 years, were included. The sample sizes varied between 50 and 121, and the disease duration ranged from 10 hours to 9 weeks. **Table 1** displays the detailed characteristics of the studies.

**Table 1:** Characteristics of included studies.

Study(Trs)	Age • T • C	Sample Size (male/female) • T • C	Course of the Disease • T • C	Intervention • T • C	Acupoints	EA parameters,Treatment Frequency and Duration • T • C	Outcomes • Major • Minor	Adverse events • T • C
**Liu L 2023**	• 56 ± 12Y • 55 ± 12Y	• 30(30/0) • 29(29/0)	• 11.0 ± 6.7H • 10.7 ± 6.6H	• Electroacupuncture +Diclofenac sodium • Diclofenac sodium	Sanyinjiao(SP6) Dadu(SP2),Taichong (LR3), Taibai(SP3), Neiting(ST44),Zusanli (ST36),Yinlingquan(SP9)	• 2Hz, Tolerated by the patient, 30 min, once • Oral medication: Diclofenac sodium, 50mg, once	• VAS • Joint Pain and Swelling Score	• None • 2 Diminished appetite
**Wu B 2021**	• 51.3 ± 5.6Y • 52.7 ± 6.1Y	• 43(30/13) • 43(31/12)	• 18.65 ± 6.48H • 20.36 ± 7.52H	• Electroacupuncture +Loxoprofen Sodium • Loxoprofen Sodium	Sanyinjiao(SP6), Taichong(LR3),Zusanli (ST36),Yinlingquan(SP9)	• 2Hz, 1mA, 30 min×1Week • Oral medication: Loxoprofen Sodiumn, 60mg, TID×1Week	• Effective rate, VAS • CRP, IL-1β	• 3 Dausea and fatigue • 4 Dausea and fatigue
**Zhong Y 2020**	• 41 ± 8Y • 41 ± 8 Y	• 41(31/10) • 41(33/8)	• 4.25 ± 0.58D • 4.31 ± 0.61D	• Electroacupuncture +Colchicine; Allopurinol; • Colchicine; Allopurinol	Sanyinjiao(SP6), Taichong(LR3),Taibai(SP3), Zusanli(ST36),Hegu(LI4), Xingjian(LR2),Pishu(BL20), Yinlingquan(SP9)	• 2/10/100 Hz,1 mA, 20min, QD× 2Weeks • Oral medication: Colchicine, first dose 1mg, then 0.5mg, BID; Allopurinol, 100mg,TID× 2Weeks	• Effectiverate, VAS, SUA • ESR, Scr,IL-1β, TNF-α, COX-2	NR
**Lan S 2018**	• 47.4 ± 8.3Y • 45.1 ± 9.2Y	• 25(20/5) • 25(21/4)	• 24.2 ± 10.2H • 25.5 ± 9.9H	• Electroacupuncture + Etoricoxib • Etoricoxib	Sanyinjiao(SP6), Zusanli(ST36),Yinlingquan (SP9), Fenglong(ST40), Quchi (LI11), Neiting(ST44Gongsun (SP4),Taixi(KI3)	• 2/100HZ, 2-4mA,Tolerated by the patient, 30min,QD×1Week • Ora medication:Etoricoxib, 120mg, QD×1Week	• Effective rate, VAS, SUA • ESR	• 1 Light-headedness • 1 Light-headedness, 1 Mild nausea,
**Wu Y 2012**	• 45.9 ± 3.9Y • 47.8 ± 4.7Y	• 34(30/4) • 32(29/3)	• 2.97 ± 1.61D • 3.12 ± 1.70D	• Electroacupuncture + Fire acupuncture; Hemorrhage therapy; • Colchicine; Allopurinol	Sanyinjiao(SP6), Zusanli(ST36),Ashipoint	• 2Hz, 30min,QD×6Days • Oral medication:Colchicine, first dose 1mg, the 0.5mg;Allopurinol, 100mg, QD×6Days	• Effective rate, VAS, SUA • ESR, CRP	NR
**Jin Z 2012**	• 38-60Y • 40-65Y	• 30(27/3) • 30(29/1)	• 1-10D • 2-12D	• Electroacupuncture + Fire acupuncture; Hemorrhage therapy; • Colchicine; Indomethacin	Sanyinjiao(SP6), Yinlingquan(SP9),Yanglingq- uan (GB34),Taibai(SP3),Taic- hong(LR3),Fenglong(ST40), Quchi (LI11),Hegu(LI4)	• Current as tolerated by the patient, 40min, QD×2Weeks • Oral medication:Colchicine first dose 1mg, then 0.5mg,TID; Indomethacin, 25mg, TID×2Weeks	• Effective rate, VAS • None	NR
**Liu Z 2010**	• 31-67Y • 30-65Y	• 32(25/7) • 33(27/6)	• 1-8W • 1-9W	• Electroacupuncture + Angelica sinensis injection; • Colchicine; Indomethacin	Sanyinjiao(SP6), Zusanli(ST36),Yinlingquan (SP9), Neiting(ST44),Taichong (LR3), Quchi(LI11),Hegu(LI4)	• Current as tolerated by the patient, 30min,QD×2Weeks • Oral medication: Colchicine first dose 1mg, then 0.5mg,TID; Indomethacin 25mg, TID×2Weeks	• Effective rate, VAS, SUA • None	• None • 9 Gastrointestinal re actions; 3 Nervous system reaction; 1 Leucopenia; 2 Rash
**Din Y 2009**	• 31-68Y • (30,70)	• 35(25/10) • 35(26/9)	• 5-11D • 5-12D	• Electroacupuncture + Indomethacin; Allopurinol; • Indomethacin; Allopurinol	Sanyinjiao(SP6), Zusanli(ST36),Ashi point	• 0.5-2mA, 30min,QD×6Days • Oral medication: Indomethacin 25mg, TID;Allopurinol, 100mg, TID×6Days	• Effective rate, VAS, SUA • None	NR
**Jin H 2009**	• 28-67Y • 30-65Y	• 34(29/5) • 33(30/3)	• 3-12D • 4-15D	• Electroacupuncture + Hemorrhage therapy; • Indomethacin; Allopurinol	Sanyinjiao(SP6), Yinlingquan(SP9),Ashi point	• 2/100Hz, Current as tolerated by the patient, 30min,QD×1Week • Oral medication:Indomethacin, 25mg, TID;Allopurinol, 100mg, TID×1Week	• Effective rate, VAS, SUA • None	NR
**He Y 2008**	• 28-67Y • 30-65Y	• 30(27/3) • 30(28/2)	• 2-13D • 3-12D	• Electroacupuncture + Trimethoprim Injection; Lidocaine injection; • Colchicine;Indomethacin	Sanyinjiao(SP6), Zusanli(ST36),Taixi(KI3),Gong- sun(SP4),Ashi point	• Current as tolerated by the patient, 20min, QD×10Days • Oral medication: Colchicine first dose 1mg, then 0.5mg,TID; Indomethacin, 25mg, TID×10Days	• Effective rate, • None	• None • 8 Gastrointestinal re-actions; 3 Nervous system reaction; 1 Leucopenia; 1 Rash
**Liu B 2008**	• 55.8 ± 8.9Y • 54.3 ± 8.4Y	• 56(51/5) • 44(40/4)	• 8.42 ± 1.91D • 8.11 ± 1.24D	• Electroacupuncture + Lidocaine injection; Prednisolone Acetate Injection; • Indomethacin; Allopurinol	Sanyinjiao(SP6), Yinbai(SP1),Zusanli(ST36),Yi- nlingquan(SP9),Yanglingquan (GB34),Fenglong(ST40), Taich- ong(LR3)	• Current as tolerated by the patient, 30min, QD×1Week • Oral medication:Indomethacin, 25mg, TID×1Week Allopurinol, 100mg,TID×1Week	• Effective rate, SUA • None	NR
**Zou R 2007**	• 32-70Y • 31-72Y	• 30(24/6) • 30(25/5)	• 4-12D • 5-13D	• Electroacupuncture + Angelica sinensis injection; • Indomethacin; Allopurinol	Sanyinjiao(SP6), Zusanli(ST36), Ashi point	• 2/100Hz, 0.5-1mA, 30min, QD× 6Days • Oral medication:Indomethacin, 25mg, TID;Allopurinol, 100mg, TID×6Days	• Effective rate, VAS, SUA • None	NR
**Zou R 2006**	• 31-70Y • 35-71Y	• 30(26/4) • 30(28/2)	• 5-13D • 3-10D	• Electroacupuncture; • Indomethacin; Allopurinol	Sanyinjiao(SP6), Zusanli(ST36), Ashi point	• 2Hz, 0.5-2mA, 30min,QD×6Days • Oral medication:Indomethacin, 25mg, TID; Allopurinol, 100mg, TID× 6Days	• Effective rate, VAS, SUA • UUA	NR
**Yin Y 2005**	• 60.3 ± 8.1Y • 62.5 ± 7.4Y	• 40(28/12) • 30(19/11)	• 6.9 ± 4.1D • 6.5 ± 3.3D	• Electroacupuncture + Indomethacin; Benzbromarone; • Indomethacin; Benzbromarone	Zusanli(ST36), Fenglong(ST40),Ashi point	• Current as tolerated by the patient, 30min, QD×6Days • Oralmedication: Indomethacin, 25mg, TID;Benzbromarone, 50mg, QD×6Days	• Effective rate, SUA • None	NR
**Zhang J 2005**	• 20-68Y • 21-67Y	• 76(71/5) • 45(43/2)	• 1-7D • 1-5D	• Electroacupuncture + Compound Dansheninjection • Allopurinol	Sanyinjiao(SP6), Yinbai(SP1),Dadun(LR1),Taich- ong (LR3),Yanglingquan(GB34), Yinlingquan(SP9)	• Current as tolerated by the patient, 30min, QD×10Days • Oral medication:Allopurinol, 200mg, TID×10Days	• Effective rate • None	NR

H,hours D,days; Y,years; W,weeks; T,treatment; C,control; NR,not recorded; QD,once a day; TID,thrice a day; VAS,visual rating scale; SUA= serum uric acid; CRP,C-reactive protein; IL,Interleukin; ESR,erythrocyte sedimentation rate; SCr,serum creatinine; UUA,urine uric acid.

### Electroacupuncture therapy interventions

In all 15 studies, electroacupuncture was added to the treatment of AGA. 3 Studies Combining Electroacupuncture and Bloodletting Therapy (31, 32, 35). Electroacupuncture was combined with oral medications in 6 studies (21, 23, 26, 27, 30, 34) and with injectable medications in 5 studies (22, 33, 36–38). In addition, 1 study used only electroacupuncture for the treatment of AGA (39).

All studies compared the effects of the electroacupuncture group with those of the oral drug group. The randomized controlled trials included in (21–23, 26, 27, 30–39) used a variety of acupoints for acupuncture treatment. The highest frequency of use was Sanyinjiao(SP6) with 14 sessions, followed by ZuSanli(ST36) with 12 sessions. Of note, seven studies used Ashi points (23, 26, 34–36, 38, 39), which are pain points for needle entry.

Ten studies compared the effects of electroacupuncture combined with drugs versus drugs alone (21–23, 26, 27, 30, 33, 34, 36–38). 4 studies compared the effect of electroacupuncture alone or in combination with non-pharmacological therapies versus drugs alone (31, 32, 35, 39). A total of 8 studies opted for electroacupuncture at a frequency of 2 Hz (21, 26, 27, 30, 32, 35, 38, 39). In 10 studies, the duration of electroacupuncture was 30 min (21–23, 26, 30, 32–35, 37–39). In 2 studies, the duration of electroacupuncture was 20 min (27, 36), and in 1 study, the duration was 40 min (31). The frequency of electroacupuncture in 14 studies was QD, with a minimum treatment period of 6 days (23, 32, 34, 38, 39) and a maximum of two weeks (27, 31, 33). Only one study performed only one electroacupuncture treatment was used to study the immediate analgesic efficacy of electroacupuncture (21).

### Control interventions

All patients received conventional western oral therapy to control acute exacerbations of gouty arthritis (21–23, 26, 27, 30–39), specifically, treatment regimens using antigout medications and NSAIDs alone or in combination. Five studies used indomethacin in combination with allopurinol (34, 35, 37–39), three studies used colchicine in combination with indomethacin (31, 33, 36), two studies used colchicine in combination with allopurinol (27, 32), and one study used indomethacin in combination with benzbromarone (23). Four studies did not combine drugs and used diclofenac sodium (21), loxoprofen sodium (30), etoricoxib (26), and one used allopurinol alone (22).

### Outcome measures

Fourteen studies reported efficacy (22, 23, 26, 27, 30–39), 11 studies assessed analgesic effect by VAS (21, 26, 27, 30–35, 38, 39), 10 studies assessed serum uric acid levels (23, 26, 27, 32–35, 37, 38), The mean date interval between measurements of VAS was 8.1 ± 4.2 d. In contrast, regarding serum uric acid levels, it was 7.3 ± 3.7 d. Two studies reported immediate analgesic effect of VAS (21, 31). Notably, only five studies reported adverse events.

### Risk of bias

**Figure 2:**
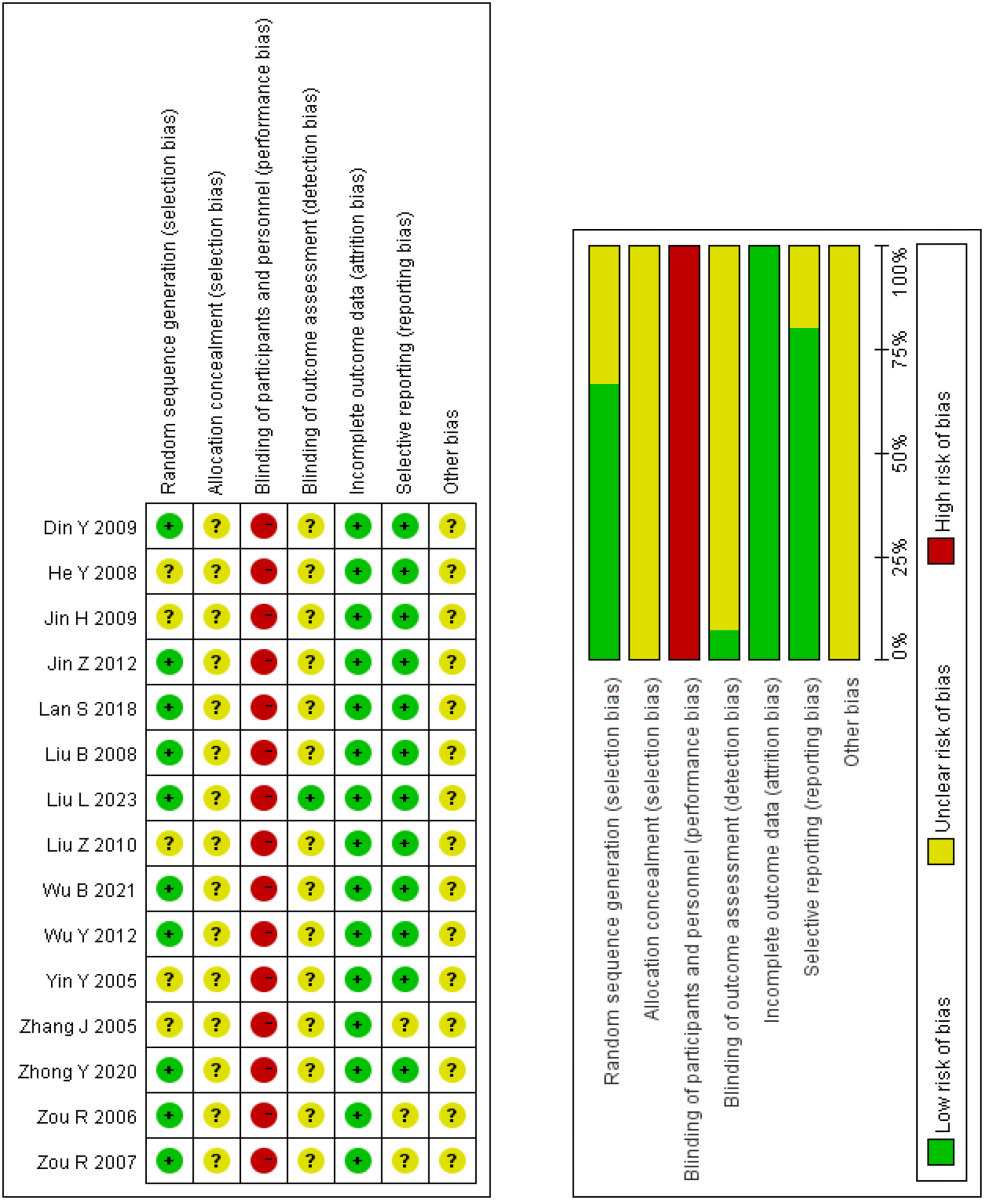
Risk of bias.

The quality of the included studies was assessed as low to moderate by the Cochrane Risk of Bias (RoB) tool V.5.1.0. **Figure 2** displays the risk of bias for each study. Ten studies used a randomized grouping method, including one randomized envelope (21), and nine used a randomized numeric table method (26, 27, 30–32, 34, 37–39), which we considered a low risk of bias for these 10 studies. The method of randomization was not specified in the remaining 5 studies. Given the specificity of electroacupuncture, blinding of electroacupuncture therapists was not feasible. Consequently, we deemed all 15 studies to have a high risk of performance bias. None of the studies discovered any missing outcomes or selective reporting, which we assessed as having a low risk of bias. Blinding of outcome assessment was only mentioned in one study (21). No other bias was explicitly discussed in any of the trials.

### Outcomes

#### Effective rate

**Figure 3:**
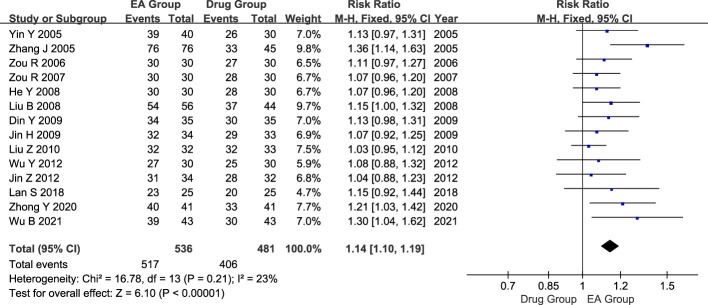
Forest plot of effective rate. Forest plot showing the comparison in effective rate for AGA between electroacupuncture and conventional medication.

There were 14 studies conducted with a total of 1017 patients suffering from AGA. Of these, 536 patients were in the electroacupuncture group, while 481 were in the control group receiving conventional western medicine. The heterogeneity test indicated a slight statistical heterogeneity across the studies. (P=0.21, I^2^ = 23%). Therefore, the meta-analysis used a fixed-effects model. The results showed that there was a significant difference in the efficacy of AGA between the electroacupuncture group and the conventional western medicine group (RR=1.14, 95% CI=1.10 to 1.19, P < 0.00001, **Figure 3**).

#### Analgesic effect (VAS)

**Figure 4:**
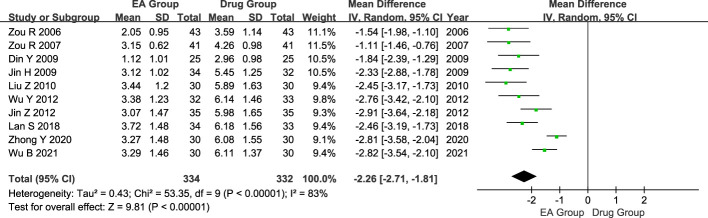
Forest plot of analgesic effect. Forest plot showing the comparison in analgesic effect for AGA between electroacupuncture and conventional medication.

Ten studies included 666 patients with AGA, 334 in the electroacupuncture group, and 332 in the conventional western medicine control group. The heterogeneity test revealed significant statistical heterogeneity among the studies. (P < 0.00001, I^2^ = 83%). Hence, a random effects model was utilized in the meta-analysis, revealing a significant difference in the pain-relieving effects between the electroacupuncture group and the conventional western medicine group. (MD = -2.26, 95% CI = -2.71 to -1.81, P < 0.00001, **Figure 4**).

#### Serum uric acid level (SUA)

**Figure 5:**
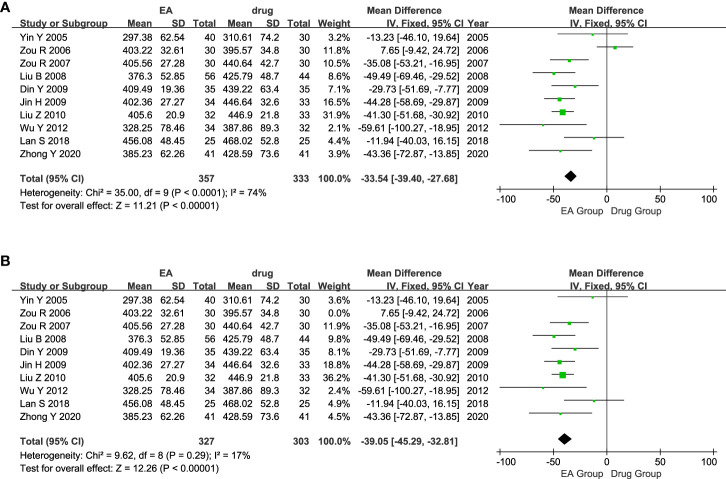
Forest plot of Serum uric acid level. **(A)** Forest plot showing the comparison in SUA level for AGA between electroacupuncture and conventional medication. **(B)** Forest plot for sensitivity analysis.

The ten studies included 690 patients with AGA, with 357 in the electroacupuncture group and 333 in the conventional western medicine control group. The heterogeneity test conducted on the ten studies indicated a significant statistical heterogeneity among them. (P < 0.0001, I^2^ = 74%). Consequently, we opted for a random effects model to analyze the data. The findings indicated a noteworthy distinction in the effects of the electroacupuncture group and the traditional western medicine group on SUA levels. (MD = -31.60, CI [-44.24, -18.96], P < 0.00001, **Figure 5A**). The sensitivity analysis results demonstrated that excluding the Zou 2006 study (31), there were 630 patients in the remaining nine studies. Among these, 327 patients belonged to the electroacupuncture group, while 303 belonged to the conventional western medicine group. Groups showed less heterogeneity (P = 0.29, I^2^ = 17%). Therefore, we re-adopted the fixed-effects model for the analysis. The results of the Meta-analysis showed that there was a significant effect of difference in the effect of SUA between the electroacupuncture group and the traditional western medicine group (MD = -39.05, CI [-45.29, -32.81], P < 0.00001, **Figure 5B**). Consistency was observed between the outcomes of the fixed-effects and random-effects models.

#### Immediate analgesic effect (VAS)

**Figure 6:**
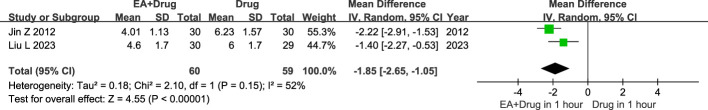
Forest plot of Immediate analgesic effect. Forest plot showing the comparison in Immediate analgesic effect for AGA between electroacupuncture plus medication and conventional medication.

119 patients with AGA were included in the two studies, 60 in the electroacupuncture plus medication group and 59 in the conventional medication group. Heterogeneity test showed moderate statistical heterogeneity between studies (P=0.15, I^2^ = 52%). The random effects model analysis revealed a statistically significant divergence in pain relief between the group receiving electroacupuncture plus medication and the group receiving only traditional Western medication within one hour. (MD = -1.85, CI [-2.65, -1.05], P < 0.00001, **Figure 6**).

#### Adverse events

**Figure 7:**
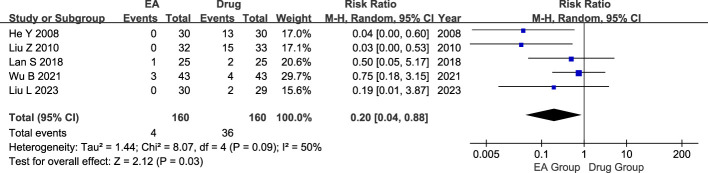
Forest plot of Adverse events. Forest plot showing the comparison in Adverse events for AGA between electroacupuncture and conventional medication.

320 patients were included in the five studies, including 160 in the electroacupuncture group and 160 in the conventional medication group. Heterogeneity test showed moderate statistical heterogeneity between studies (P=0.09, I^2^ = 50%). Significant heterogeneity was observed in the incidence of adverse events between the electroacupuncture and conventional drug groups according to the results of the random-effects model analysis. (RR=0.20, 95% CI=0.04 to 0.88, P=0.03, **Figure 7**).

### Subgroup analysis

We performed subgroup analyses for three categorical indicators: A) Different electroacupuncture combinations. B) Sample size. C) Electroacupuncture frequency. Specific results are detailed in **Table 2**.

**Table 2:** Subgroup analysis results.

Subgroup		efficiency rate				VAS				SUA			
		Study	RR [95%CI]	*P* value	*I* ^2^	Study	MD[95%CI]	*P* value	*I* ^2^	Study	MD [95%CI]	*P* value	*I* ^2^
Total		14	1.14 [1.10-1.19]	0.0001	23%	10	-2.26 [-2.71,-1.81]	0.0001	83%	10	-31.60 [-44.24,-18.96]	0.0001	74%
Interventions	• non-pharmaceutical	4	1.07 [0.99-1.17]	0.08	0%	4	-2.49 [-2.82,-2.16]	0.00001	0%	3	-29.97 [-71.22,-11.29]	0.15	92%
	• combination of drugs	10	1.17 [1.11-1.23]	0.00001	49%	6	-2.11 [-2.74,-1.49]	0.0001	87%	7	-35.88 [-44.66,-27.10]	0.0001	24%
Sample size	• n<40	9	1.08 [1.03-1.14]	0.002	0%	8	-2.50 [-2.77,-2.23]	0.00001	23%	3	-29.55 [-45.32,-13.77]	0.0001	80%
	• n≥40	5	1.23 [1.14-1.33]	0.00001	1%	2	-1.30 [-1.72,-0.88]	0.0001	55%	7	-38.33 [-58.73,-17.94]	0.18	42%
EA frequency	• 2Hz	3	1.13 [1.00-1.26]	0.05	31%	3	-2.19 [-2.93,-1.45]	0.00001	81%	2	-23.34 [-89.05,42.37]	0.49	89%
	• >2Hz	11	1.11 [1.06-1.17]	0.00001	27%	7	-2.30 [-2.92,-1.68]	0.00001	86%	8	-37.76 [-45.20,-30.33]	0.0001	19%

EA, electroacupuncture; RR, risk rate; MD, mean difference; VAS, visual assessment scale; SUA, serum uric acid; CI, confidence interval.

#### Subgroup analysis of efficiency

##### Different electroacupuncture combinations

No significant heterogeneity was found among the 4 studies (I2 = 0%) in the subgroup analysis comparing drug-free electroacupuncture to medication. The fixed-effects model analysis demonstrated no significant difference in efficacy between drug-free electroacupuncture and drug treatment for AGA (RR=1.07, 95% CI=0.99 to 1.17, P=0.08). A mild statistical heterogeneity was observed among the 10 studies (I2 = 49%) comparing electroacupuncture plus medication to conventional medication in the subgroup analysis. The results of the fixed-effects model revealed a significant difference in the efficacy of electroacupuncture plus medication and conventional medication for AGA (RR=1.17, 95% CI=1.11 to 1.23, P<0.00001).

##### Sample size

In the subgroup analysis with a sample size of less than 40, no significant heterogeneity was observed among the 9 studies (I^2^ = 0%). The analysis using a fixed-effects model revealed a significant difference in efficacy between the electroacupuncture group and the conventional drug group for AGA (RR=1.08, 95% CI [1.03-1.14], P=0.002). In the subgroup analysis with a sample size of 40 or more, the heterogeneity test indicated mild statistical heterogeneity among the 5 studies (I^2^ = 1%). The results from the fixed-effects model analysis showed a significant difference in efficacy between the electroacupuncture group and the conventional medication group for AGA (RR=1.23, 95% CI [1.14-1.33], P<0.00001).

##### Electroacupuncture frequency

In the subgroup analysis of electroacupuncture frequency of 2 Hz, mild heterogeneity was noted among the three studies (I^2^ = 31%). The fixed-effects modeling analysis demonstrated a significant difference in efficacy between the electroacupuncture and conventional medication groups for AGA (RR = 1.13, 95% CI [1.00-1.26], P = 0.05). During the subgroup analysis of electroacupuncture frequency exceeding 2 Hz, the heterogeneity test indicated mild statistical heterogeneity across the eleven studies (I^2^ = 27%). The results obtained from the fixed-effects model revealed a significant difference in efficacy between the electroacupuncture group and the conventional medication group for AGA (RR = 1.11, 95% CI [1.06-1.17], P<0.00001).

#### Subgroup analysis of pain visualization score (VAS)

##### Different electroacupuncture combinations

In the subgroup analysis comparing drug-free electroacupuncture and medication, no significant heterogeneity was observed among the four studies (I^2^ = 0%). The fixed-effects model analysis revealed a significant difference in pain efficacy between drug-free electroacupuncture and medication in patients with AGA (MD= -2.49, 95% CI [-2.82 to -2.16], P<0.0001). In the subgroup analysis comparing electroacupuncture plus medication and conventional medication, a high degree of statistical heterogeneity was noted among the six studies (I^2 = ^87%). The results of the random-effects model showed a significant difference in pain efficacy between the electroacupuncture plus medication group and the conventional medication group in patients with AGA (MD= -2.11, 95% CI [-2.74 to -1.49], P < 0.0001).

##### Sample size

In the subgroup analysis with a sample size of less than 40, mildly significant heterogeneity was observed among the 8 studies (I^2^ = 23%). The analysis using a fixed-effects model revealed a significant difference in efficacy between the electroacupuncture group and the conventional medication group for AGA (MD= -2.50, 95% CI [-2.77 to -2.23], P<0.00001). Meanwhile, in subgroup analyses with sample sizes of 40 or more, a heterogeneity test demonstrated moderate statistical heterogeneity between the 2 studies (I^2^ = 55%). The results obtained from the random-effects model analysis showed a significant difference in efficacy between the electroacupuncture group and the conventional medication group for AGA (MD= -1.30, 95% CI [-1.72 to -0.88], P < 0.0001).

##### Electroacupuncture frequency

In the subgroup analysis of electroacupuncture frequency = 2 Hz, there was a high degree of heterogeneity among the 3 studies (I^2^ = 81%). The random-effects model analysis revealed a significant difference in efficacy between the electroacupuncture group and the conventional medication group for AGA (MD = -2.19, 95% CI [-2.93 to -1.45], P < 0.00001). For studies with an electroacupuncture frequency of 2 Hz, the heterogeneity test showed a high degree of statistical heterogeneity among the seven studies (I^2^ = 86%). The results of the random-effects model indicated a significant difference in the efficacy of the electroacupuncture group and the conventional drug group on AGA (MD = -2.30, 95% CI [-2.92 to -1.68], P < 0.00001).

#### Subgroup analysis of serum uric acid levels

##### Different electroacupuncture combinations

In the subgroup analysis of drug-free electroacupuncture and medication, there was a high degree of heterogeneity among the three studies (I^2^ = 92%). The random effects model analysis showed no significant difference in drug-free electroacupuncture and drug treatment effects on serum uric acid levels (MD= -29.97, 95% CI [-71.22 to -11.29], P=0.15). In the subgroup analysis of electroacupuncture plus medication versus conventional medication, the heterogeneity test showed mild statistical heterogeneity among the seven studies (I^2^ = 24%). The results using the fixed-effects model indicated a significant difference in the effect of electroacupuncture plus drugs on serum uric acid levels compared to conventional medications (MD= -35.88, 95% CI [-44.66 to -27.10], P<0.0001).

##### Sample size

In the subgroup analysis involving a sample size of less than 40, a highly significant heterogeneity was observed among the three studies (I^2^ = 80%). Results from the analysis using a random-effects model showed a considerable difference in efficacy between the electroacupuncture group and the conventional drug group for AGA (MD= -29.55, 95% CI [-45.32 to -13.77], P=0.0001).In the subgroup analysis with a sample size of 40, the heterogeneity test revealed mild statistical heterogeneity between the seven studies (I^2^ = 42%). The fixed-effects modeling results indicated no significant difference in efficacy between the electroacupuncture group versus the conventional medication group for AGA (MD= -38.33, 95% CI [-58.73 to -17.94], P=0.18).

##### Electroacupuncture frequency

The two studies showed significant heterogeneity in the subgroup analysis of electroacupuncture frequency = 2 Hz (I^2^ = 89%). When analyzing the data using a random-effects model, no significant difference in efficacy was found between the electroacupuncture and conventional medication groups for AGA (mean difference = -23.34, 95% confidence interval [-89.05 to 42.37], p= 0.49). However, in the subgroup analysis of electroacupuncture frequency >2 Hz, there was mild statistical heterogeneity among the eight studies (I^2^ = 19%). The results obtained from the fixed-effects model demonstrated a significant difference in efficacy between the electroacupuncture group and the conventional medication group for AGA (mean difference = -37.76, 95% confidence interval [-45.20 to -30.33], p < 0.0001).

### Sensitivity analysis

**Figure 8:**
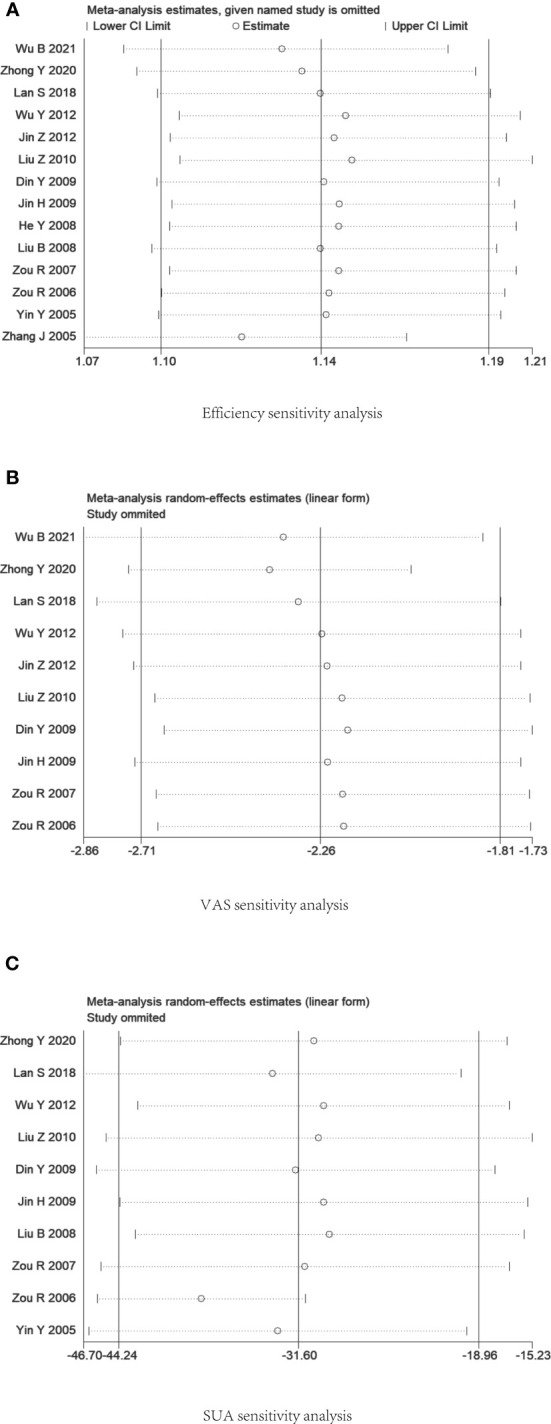
Plot of sensitivity analysis. **(A)** Efficiency rate. **(B)** Visual Rating Scale. **(C)** Serum uric acid level.

Sensitivity analysis of the data was performed using Stata V12.0 software. The results suggested that the data sensitivity in each group was generally stable. The results of the sensitivity analysis of serum uric acid level suggested that Zou R 2006 was the main source of heterogeneity. **Figure 8** presents the outcomes of the sensitivity analysis.

### Publication bias

**Figure 9:**
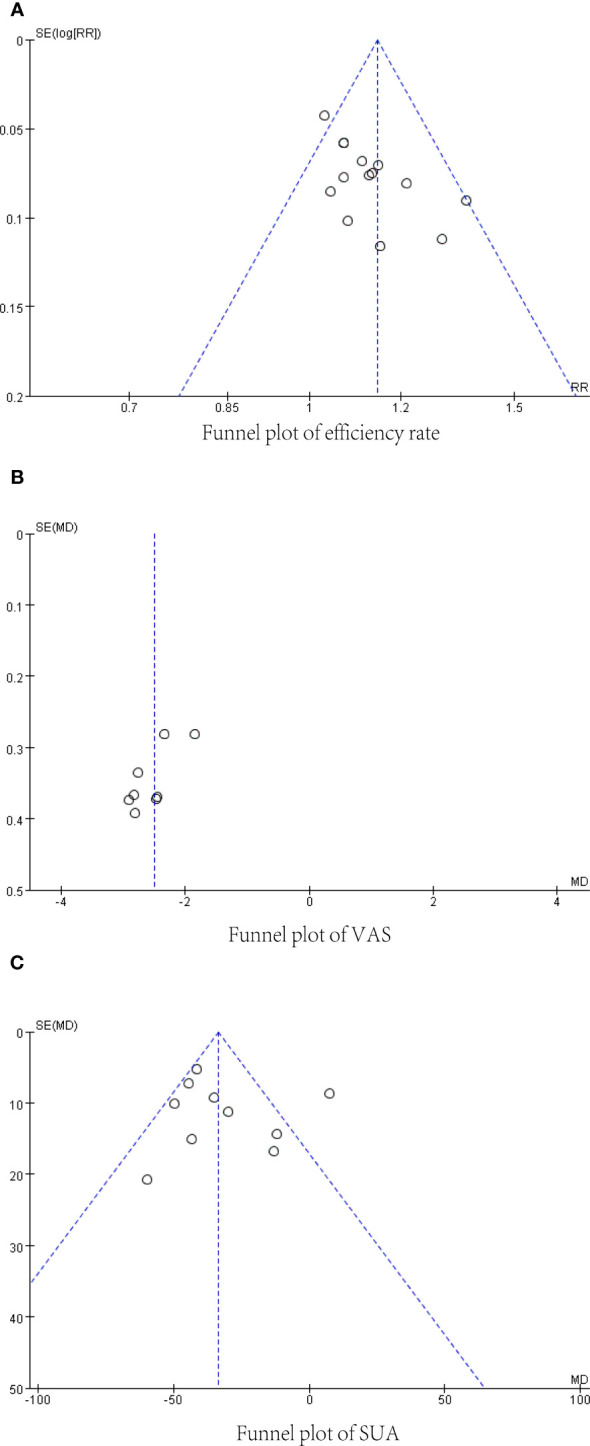
Funnel plot. **(A)** Efficiency rate. **(B)** Visual Rating Scale. **(C)** Serum uric acid level.

Publication bias for each main indicator was visually analyzed using funnel plots. There was a slight asymmetry in the funnel plot for validity (**Figure 9A**), Begg’s Test, P=0.055, and Egger’s Test, P=0.009, suggesting that some publication bias may exist. The funnel plot of the pain visual rating scale is detailed in (**Figure 9B**), Begg’s Test, P=0.02, Egger’s Test, P<0.001, suggesting that there may be some publication bias. There was no significant asymmetry in the funnel plot of serum uric acid level (**Figure 9C**), Begg’s Test, P=0.474, Egger’s Test, P=0.632, suggesting that publication bias is insignificant. The small number of studies prevented the evaluation of immediate analgesic effects and adverse events using funnel plots, possibly causing some publication bias.

### GRADE evaluation

This study’s quality of evidence assessment was processed considering five downgrading factors (i.e., risk of bias, inconsistency, indirectness, imprecision, and publication bias). This study’s quality of evidence assessment was handled considering five downgrading factors (i.e., risk of bias, inconsistency, indirectness, imprecision, and publication bias). Of all 15 studies, 10 mentioned the randomization allocation method, and 5 studies did not specify the randomization method. Only 1 of the 15 studies mentioned blinding of the measurers, so the risk of bias for all studies was reduced by 1 level. For 14 studies on effectiveness, funnel plots, and statistical tests suggested publication bias, and publication bias was downgraded by 1 level. For the 10 studies on analgesic effect, there was a high degree of heterogeneity between studies, so inconsistency was downgraded by 1. Funnel plots and statistical tests suggested the presence of publication bias, and publication bias was downgraded by 1. The 10 studies on lowering serum uric acid levels had high inter-study heterogeneity, but we found the source of heterogeneity and did not downgrade. For the 2 studies on immediate analgesic effect, inconsistency and publication bias were each downgraded by 1 grade because of high heterogeneity and a small number of RCTs. Regarding adverse events, the number of RCTs was small, and publication bias was downgraded by 1 grade. Overall, the level of evidence for lowering serum uric acid levels was intermediate, the level of evidence for effectiveness and incidence of adverse events was low, and the level of evidence for analgesic effect and immediate analgesic effect was very low. Detailed results are shown in **Table 3**.

**Table 3:** Overall evidence GRADE quality rating of seven included studies.

Outcomes	Number of Studies	Study Design	Sample SizeTC	Degradation Factors					Effect Size	Evidence Grade
				Risk of Bias	Inconsistency	Indirect	Imprecision	Publication Bias		
Effective rate	14	RCT	• T536 • C481	-1^a^				-1^c^	(RR= 1.14, 95% CI= 1.10 to 1.19)	low
Analgesic effect(VAS)	10	RCT	• T334 • C332	-1^a^	-1^b^			-1^c^	(MD= -2.26, 95% CI= -2.71 to -1.81)	very low
SUA	10	RCT	• T357 • C333	-1^a^					(MD= -31.60, 95%CI= -44.24 to -18.96)	middle
Immediate analg-esic effect(VAS)	2	RCT	• T60 • C59	-1^a^	-1^b^			-1^c^	(MD= -1.85, 95%CI= -2.65 to -1.05)	very low
Adverse events	5	RCT	• T160 • C160	-1^a^				-1^c^	(RR= 0.20, 95% CI= 0.04 to 0.88)	low

T, treatment; C, control; RCT, randomized controlled trial; RR, risk ratio; CI, confidence interval; VAS,visual rating scale; SUA= serum uric acid.

a The design of the trial has a large bias in randomization, allocation concealment, or blinding.

b The credible interval overlaps less, the P-value of the heterogeneity test is small, and the I of the combined results is large.

c Statistical results indicating publication bias or small sample size suggesting possible publication bias.

## Discussion

### Main results

This systematic review examined 15 randomized controlled trials that investigated the use of electroacupuncture as a treatment for AGA. As far as we know, this is the initial systematic review to assess the effectiveness of electroacupuncture for AGA, and it followed the meta-analysis guidelines outlined by the Preferred Reporting Items for Systematic Reviews and Meta-Analyses.

Acute exacerbations of gouty arthritis have been described as the most troublesome condition by gout patients (8). The current meta-analysis suggests that electroacupuncture and conventional treatments have comparable efficacy and safety in targeting painful symptoms in patients with AGA. In addition, subgroup analyses of types of interventions performed considering the effect of heterogeneity showed no significant difference between electroacupuncture without the combination of medication and medication alone in terms of efficiency and analgesic effect. Interestingly, electroacupuncture combined with medication showed better efficacy, analgesic effect, and serum uric acid reduction than medication alone. However, in a subgroup analysis of the effect of non-pharmacological electroacupuncture versus drug therapy on serum uric acid levels, electroacupuncture-only therapy (39) demonstrated the opposite results, possibly as a result of electroacupuncture alone. It is noteworthy that electroacupuncture combined with drug therapy was better than drug therapy alone in terms of immediate analgesic effect.

After summarizing the electroacupuncture stimulation methods used in the studies, the results showed that the set stimulation time was concentrated at 20-40 minutes, with 12 studies setting the time at 30 minutes. Regarding the choice of acupuncture points for electroacupuncture stimulation, Sanyinjiao (SP6, located on the medial calf above the tip of the inner ankle, behind the medial border of the tibia) and Zusanli (ST36, located on the anterolateral calf, one transverse finger away from the anterior border of the tibia) appeared most frequently and were the main acupuncture points used for needling. Overall, the layout of electroacupuncture stimulation mainly focused on the anterolateral and anteromedial sides of the calf. It is important to note that the current level of electroacupuncture stimulation is clinically adjusted to suit the patient’s tolerance level.

A study published by Lu et al. in 2016 (40) aligns with our findings. This study conducted a meta-analysis of seven studies that used visual analog scale scores to evaluate AGA. The results indicated that acupuncture demonstrated greater pain relief in AGA patients compared to conventional medication. (pooled MD = -1.92; 95% CI = -2.87 to -0.96). In conclusion, electroacupuncture is an extremely viable complementary alternative treatment for individuals with AGA who cannot undergo primary treatment due to contraindications, those who do not want to take systemic medications or those who prioritize temporary pain alleviation.

In our study, the heterogeneity of the ten studies on AGA pain was high. (P < 0.00001, I^2^ = 83%), and we performed a subgroup analysis, which showed no significant heterogeneity among the four studies on the nonpharmacological electroacupuncture group and the pharmacological group alone (P = 0.76, I^2^ = 0%). Then, in six studies comparing the efficacy of electroacupuncture in combination with drugs versus conventional drugs, statistically significant heterogeneity was found. (P<0.00001, I^2^ = 87%), for which we hypothesized that the source of heterogeneity might be related to different drug combinations. In addition, we found the source of heterogeneity in the study of serum uric acid levels by sensitivity analysis (P < 0.0001, I^2^ = 74%). When the study of Zou 2006 was excluded, there was less heterogeneity among the studies (P = 0.29, I^2^ = 17%). We believe this may be because the study used electroacupuncture alone.

Current studies have shown that the pathogenesis of gout is relatively clear (13, 14, 41). Formation and deposition of monosodium urate (MSU) crystals occur as a result of hyperuricemia (serum uric acid levels >7 mg/l (420μmol/l). MSU crystals trigger inflammatory vesicles to mediate the production of IL-1β, which mediates inflammation and is a key aspect of gouty arthritis. The release of IL-1β through the inflammasome activates a significant inflammatory response, causing vasodilation and the quick arrival of neutrophils at the location where crystals are deposited. This results in an acute inflammatory attack that induces pain-related symptoms in patients (42). Nociception results from transmitting unmyelinated, slow, conducting C nerve fibers from the periphery to the brain’s center. Both peripheral and central sensations of pain are lessened by the release of endogenous opioids. The release of endogenous opioids from lymphocytes, monocytes/macrophages, and granulocytes is triggered by electroacupuncture, activating receptors on peripheral nerve endings. This activation then works to suppress nociception (43, 44).

In treating gouty arthritis, we are concerned with the patient’s need for immediate analgesia. We also expect to reduce excess uric acid storage, leading to the treatment and management of gout through long-term reduction of serum uric acid concentration. Our study showed that when electroacupuncture and medication were used together, there was a more significant decrease in serum uric acid than alone. Nevertheless, the specific mechanism responsible for this effect on uric acid levels remains unclear and needs additional investigation.

The included studies showed moderate to high risk of bias and heterogeneity in the meta-analysis, which usually puts the level of evidence low or very low. We can only give a weak recommendation for electroacupuncture for AGA based on the available GRADE evidence from this study. However, our results remain positive for the treatment of AGA. In future randomized controlled trials, consider more logically designed electroacupuncture protocols, given that the electroacupuncture technique allows for partial standardization. This would enable the acquisition of high-quality, evidence-based results. In summary, there is no comprehensive understanding of the mechanisms involved in electroacupuncture for treating AGA, and additional research is necessary. The study’s level of evidence is low, so the results should be interpreted carefully. Further validation of our findings requires larger sample sizes and more well-designed RCTs.

### Limitations

First, the completion of the RCTs in this study took place solely in China, without any multicenter trials. This could potentially impact the overall quality of evidence presented in this study.

Secondly, electroacupuncture, as a distinctive complementary alternative medicine therapy, presents difficulties when implementing blinding in research. Moreover, developing precise specifications regarding stimulation intensity and acupoint selection for electroacupuncture methods remains challenging. Therefore, the efficacy of electroacupuncture interventions may be influenced by bias.

Third, according to the ACR guidelines, medication selection should be determined by the number of joints affected and the severity of pain. It is recommended that identified pharmacologic therapies for reducing uric acid levels be continued without breaks. Nevertheless, certain studies included in our analysis only provided first-line treatment options such as NSAIDs, corticosteroids, and colchicine, making no mention of the management of ULT. This omission may potentially influence the interpretation of the findings.

Fourth, there may be publication bias regarding trial design and outcome evaluation. We note that the outcome evaluations for both efficiency and analgesia used manually evaluated scales, and the possibility exists that this ultimately led to publication bias.

### Implications for practice and research

The involvement of electroacupuncture in managing various types of pain is gaining popularity, and there is a need to understand its potential use in managing inflammatory pain better. The data we have indicates that electroacupuncture is effective in treating AGA. Furthermore, combining electroacupuncture with medication improves the analgesic and serum uric acid-lowering effects, leading to immediate pain relief. These findings will offer clinicians more treatment options to consider. Furthermore, we recommend that patients consider electroacupuncture as a treatment for AGA due to the positive outcomes observed in the analyzed data. At the same time, alternative options might have shown comparatively weaker results.

In summary, the mechanism of electroacupuncture for AGA still needs to be fully understood, and further studies are required to clarify it. Interpretations of the results should be approached with caution due to the poor quality of the studies included. Studying future studies encourages additional randomized controlled trials with more rigorous designs and larger sample sizes.

## Conclusion

Research has shown that electroacupuncture has advantages in treating acute gouty arthritis. Furthermore, combining electroacupuncture with conventional medication has proven to provide superior pain relief and reduce uric acid levels compared to using drugs alone. Based on the current evidence, we recommend electroacupuncture therapy with Sanyinjiao and Zusanli as the main acupoints in treating AGA, with appropriate stimulation of the Ashi point for a 30-minute timeframe. Conducting additional well-designed multicenter clinical trials with larger sample sizes and more extended treatment periods is essential to validate and consolidate these findings.

## Data availability statement

The original contributions presented in the study are included in the article/**Supplementary Material**. Further inquiries can be directed to the corresponding author.

## Author contributions

ZN: Formal analysis, Investigation, Software, Writing – original draft, Writing – review & editing, Conceptualization, Data curation, Methodology. QX: Methodology, Writing – review & editing, Conceptualization, Formal analysis, Investigation. ZX: Formal analysis, Software, Writing – review & editing. KK: Data curation, Writing – review & editing. BY: Data curation, Writing – review & editing. DP: Funding acquisition, Project administration, Supervision, Writing – review & editing.
