# Supplementary material for: Electroacupuncture for acute gouty arthritis: a systematic review and meta-analysis of randomized controlled trials
Source: Front Immunol. 2024 Jan 4;14:1295154. doi: 10.3389/fimmu.2023.1295154 (PMC10794621; doi:10.3389/fimmu.2023.1295154)
Supplement: Supplementary file 1 [file DataSheet_1.docx]

| **Section and Topic** | **Item #** | **Checklist item** | **Location where item is reported** |
| --- | --- | --- | --- |
| **TITLE** | | |  |
| Title | 1 | Electroacupuncture for acute gouty arthritis：a systematic review and meta-analysis of randomized controlled trials | 1 |
| **ABSTRACT** | | |  |
| Abstract | 2 | Acute gouty arthritis (AGA) is a metabolic disorder in which recurrent episodes of pain can severely affect the quality of life of gout sufferers. Electroacupuncture (EA) is a non-pharmacologic therapy. The aim of this systematic review was to assess the efficacy and safety of electroacupuncture in the treatment of acute gouty arthritis. We searched eight Chinese and English databases from inception to July 30, 2023, and a total of 242 studies were retrieved. Finally, 15 randomized controlled trials (n=1076) were included in a meta-analysis using Review Manager V.5.4.1. meta-analysis results included efficacy rate, visual rating scale (VAS) for pain, serum uric acid level (SUA), immediate analgesic effect, and incidence of adverse events. Electroacupuncture (or combined non-pharmacologic) treatment of AGA was significantly different from treatment with conventional medications (RR = 1.14, 95% confidence interval CI = 1.10 to 1.19, P < 0.00001). The analgesic effect of the electroacupuncture group was superior to that of conventional western drug treatment (MD = -2.26, 95% CI = -2.71 to -1.81, P < 0.00001). The electroacupuncture group was better in lowering serum uric acid than the conventional western drug group (MD =-31.60, CI -44.24 to -18.96], P < 0.00001). In addition, electroacupuncture combined with western drugs had better immediate analgesic effect than conventional western drug treatment (MD = -1.85, CI -2.65 to -1.05, P < 0.00001). Five studies reported adverse events in the electroacupuncture group and the drug group, including 19 cases of gastrointestinal symptoms and 6 cases of neurological symptoms (RR = 0.20, 95% CI = 0.04 to 0.88, P = 0.03). | 1 |
| **INTRODUCTION** | | |  |
| Rationale | 3 | Current medical treatment options still have limitations in treating acute gouty arthritis. | 2 |
| Objectives | 4 | To find out the effectiveness and safety of electroacupuncture in the treatment of acute gouty arthritis | 2 |
| **METHODS** | | |  |
| Eligibility criteria | 5 | Studies that fulfilled the given criteria were considered suitable for inclusion:1) the study was conducted in individuals with a confirmed diagnosis of AGA; 2) the study design was an RCT; 3) Electroacupuncture was the main therapy, either alone or in conjunction with other approaches.; and 4) The language was restricted to only English or Chinese. Exclusion criteria were: 1) animal studies, case reports, self-controlled, non-randomized controlled trials 2) repeated published studies. | 3 |
| Information sources | 6 | We searched nine databases electronically and manually for all electroacupuncture randomized controlled trials of acute gouty arthritis, from the beginning of the database until July 31, 2023, by searching the databases. The databases included PubMed, Web of Science, Embase, Cochrane Library, China Knowledge Network (CNKI), China Biomedical Literature Database (CBM), Chinese Scientific Journal Database (VIP), and Wanfang. | 3 |
| Search strategy | 7 | Medical subject headings (MeSH) and keywords were utilized to search for the articles. (e.g., acute gouty arthritis, electroacupuncture, and randomized controlled trials) combined with Boolean logic operators. The electronic search included the utilization of the subsequent search terms: (Gouty Arthritis [MeSH Terms] OR Gouty Arthritides [Title/Abstract] OR Acute Gouty Arthritis [Title/Abstract] OR Gouts [Title/ Abstract]) AND (Electroacupuncture [MeSH Terms] OR Electroacupuncture therapy [Title/Abstract] OR Electroacupuncture treatment [Title/Abstract] OR Electroacupuncture [Title/Abstract]) AND (randomized controlled trial [Title/Abstract]). We adapted the search terms of different databases to suit their search criteria (Supplementary Appendix). | 3 |
| Selection process | 8 | Two reviewers (ZCN and QWX) independently reviewed the screening results. The first review involved examining the title, abstract, and keywords. Afterwards, a thorough examination of the full text of potential studies that matched the inclusion criteria was conducted. In the event of any disagreements that occur during this process, a resolution will be reached through consultation by a third reviewer. (DZP). | 4 |
| Data collection process | 9 | The following information will be obtained separately from the included RCTs by our researchers (ZCN and QWX), using a pre-designed extraction scale in an independent manner: first author, date of publication, sample size, age, duration of the disease, intervention, acupoints, frequency and duration of treatments, outcomes, efficacy, and adverse effects. Efforts will be made to reach out to the authors in case there is any missing or unclear information in the Randomized Control Trial (RCT). In case of any disagreements occurring during this process, they will be resolved either through consultation or by involving a third reviewer (DZP). | 4 |
| Data items | 10a | The following information will be obtained separately from the included RCTs by our researchers (ZCN and QWX), using a pre-designed extraction scale in an independent manner: first author, date of publication, sample size, age, duration of the disease, intervention, acupoints, frequency and duration of treatments, outcomes, efficacy, and adverse effects. Efforts will be made to reach out to the authors in case there is any missing or unclear information in the Randomized Control Trial (RCT). In case of any disagreements occurring during this process, they will be resolved either through consultation or by involving a third reviewer (DZP). | 4 |
|  | 10b | Participant and intervention characteristics are presented in Table 1 | 7 |
| Study risk of bias assessment | 11 | In order to assess the risk of bias, we will utilize the risk of bias tool in the Cochrane Handbook V.5.1.0. Each included study was independently assessed by two researchers (KLK and ZHX). Based on the aforementioned tool, the risk of bias was eventually classified as low, high, or unclear. In case of any discrepancies, they were resolved through discussion involving the third author. (DZP). | 4 |
| Effect measures | 12 | When there was no significant heterogeneity in the data, it was analyzed using a fixed effects model. Conversely, a random effects model was used in cases where there was significant heterogeneity between groups. Descriptive analysis methods were recommended in situations where quantitative analysis was not feasible. Effectiveness is a dichotomous variable, with only effectiveness and ineffectiveness as indicators. | 4 |
| Synthesis methods | 13a | Initially, a total of 289 studies were included, and no extra articles were identified through manual search. Following the assessment by two independent reviewers (ZCN and QWX), 15 RCTs were finally included in the study, [21-23, 27, 28, 31-40] totaling 1076 subjects. Fig 1 displays the study selection procedure. All of the 15 trials analyzed were single-center RCTs conducted in China, and they were published in English and Chinese within the timeframe of 2005-2023. one of the studies was a master's thesis in 2018 [27]. In the 15 trials, a total of 1076 patients with AGA, ranging in age from 20 to 72 years, were included. The sample sizes varied between 50 and 121, and the duration of the disease ranged from 10 hours to 9 weeks. Table 1 displays the detailed characteristics of the studies. | 5 |
|  | 13b | The Visual Assessment Scale (VAS) for pain was a manual entry. To enhance comparability and eliminate baseline error, we calculated the mean difference between the pre-treatment VAS values and the post-treatment VAS values in each study. The mean difference in VAS decrease after treatment compared to before treatment was used to compare the statistical differences between the treatment and control groups. Serum uric acid levels in each study were obtained by testing in hospital laboratories, and the post-treatment test values of the test and control groups were directly selected as statistical parameters. The measurement for data on continuous variables was conducted using the mean difference (MD) with a 95% confidence interval [CI]. For data on dichotomous variables, the outcomes were measured using the risk ratio (RR) with a 95% CI. | 4 |
|  | 13c | Meta-analysis results are presented using forest plots and publication bias is presented using funnel plots |  |
|  | 13d | The analysis of the data was carried out using Review Manager version V.5.4.1. When there was no significant heterogeneity in the data, it was analyzed using a fixed effects model. Conversely, a random effects model was used in cases where there was significant heterogeneity between groups. Descriptive analysis methods were recommended in situations where quantitative analysis was not feasible. | 4 |
|  | 13e | Heterogeneity was assessed using Higgins test by calculating I^2^, and values of I^2^ greater than 50% could be regarded as indicating significant heterogeneity. Potential reasons for the heterogeneity were examined through sensitivity and subgroup analyses as follow: 1) Different electro-acupuncture combinations and 2) Sample size and 3) Frequency of electroacupuncture.  If there were more than 10 eligible studies, funnel plots were utilized for the assessment of publication bias, while Egger's test and Begg's test were employed to verify the presence of publication bias. | 5 |
| Reporting bias assessment | 14 | If there were more than 10 eligible studies, funnel plots were utilized for the assessment of publication bias, while Egger's test and Begg's test were employed to verify the presence of publication bias. | 5 |
| Certainty assessment | 15 | The Grading of Recommendations, Assessment, Development and Evaluation (GRADE) was used to evaluate the level of evidence for each outcome. The level of evidence was categorized as high, moderate, low, or very low. The evaluation of the level of evidence considered several domains, including risk of bias, imprecision, inconsistency, indirectness, publication bias, large magnitude of effect, dose-response, and confounding. The criteria set by the GRADE group were used to assess these domains. | 5 |
| **RESULTS** | | |  |
| Study selection | 16a | See Figure 1 for details |  |
|  | 16b | See Figure 1 for details |  |
| Study characteristics | 17 | See table 1 for details | 7 |
| Risk of bias in studies | 18 | See Figure 2 for details |  |
| Results of individual studies | 19 | See Figure 3-9, Table 2-3 for details. |  |
| Results of syntheses | 20a | Publication bias for each of the main indicators was visually analyzed using funnel plots. There was a small asymmetry in the funnel plot for validity (Fig 9A), Begg's Test, P=0.055, and Egger's test, P=0.009, suggesting that some publication bias may exist. The funnel plot of pain visual rating scale is detailed in (Fig 9B), Begg's Test, P=0.02, Egger's test, P＜0.001, suggesting that there may be some publication bias. There was no significant asymmetry in the funnel plot of serum uric acid level (Fig 9C), Begg's Test, P=0.474, Egger's test, P=0.632, suggesting that publication bias is not significant. The small number of studies prevented the evaluation of immediate analgesic effects and adverse events using funnel plots, possibly causing some publication bias. | 14 |
|  | 20b | See outcome for details | 9-12 |
|  | 20c | The sensitivity analysis results demonstrated that excluding the Zou 2006 study [31], there were a total of 630 patients in the remaining nine studies. Among these, 327 patients belonged to the electroacupuncture group while 303 patients belonged to the conventional western medicine group. Groups showed less heterogeneity (P = 0.29, I^2^ = 17%). Therefore, we re-adopted the fixed-effects model for the analysis, and the results of Meta-analysis showed that there was a significant effect of the difference in the effect of SUA between the electroacupuncture group and the traditional western medicine group (MD = -39.05, CI [-45.29, -32.81], P < 0.00001, Fig 5B). Consistency was observed between the outcomes of the fixed-effects and random-effects models. | 9 |
|  | 20d | Sensitivity analysis of the data was performed using Stata V12.0 software, and the results suggested that the sensitivity of the data in each group was generally stable, and the results of the sensitivity analysis of serum uric acid level suggested that Zou R 2006 was the main source of heterogeneity. Fig 8 presents the outcomes of the sensitivity analysis. | 14 |
| Reporting biases | 21 | Publication bias for each of the main indicators was visually analyzed using funnel plots. There was a small asymmetry in the funnel plot for validity (Fig 9A), Begg's Test, P=0.055, and Egger's test, P=0.009, suggesting that some publication bias may exist. The funnel plot of pain visual rating scale is detailed in (Fig 9B), Begg's Test, P=0.02, Egger's test, P＜0.001, suggesting that there may be some publication bias. There was no significant asymmetry in the funnel plot of serum uric acid level (Fig 9C), Begg's Test, P=0.474, Egger's test, P=0.632, suggesting that publication bias is not significant. The small number of studies prevented the evaluation of immediate analgesic effects and adverse events using funnel plots, possibly causing some publication bias. | 14 |
| Certainty of evidence | 22 | The quality of evidence assessment in this study was processed considering five downgrading factors (i.e., risk of bias, inconsistency, indirectness, imprecision, and publication bias). The quality of evidence assessment of this study was handled considering five downgrading factors (i.e., risk of bias, inconsistency, indirectness, imprecision, and publication bias). Of all 15 studies, 10 mentioned the randomization allocation method and 5 studies did not specify the randomization method. And only 1 of the 15 studies mentioned blinding of the measurers, so the risk of bias for all studies was reduced by 1 level. For 14 studies on effectiveness, funnel plots and statistical tests suggested publication bias, and publication bias was downgraded by 1 level. For the 10 studies on analgesic effect, there was a high degree of heterogeneity between studies, so inconsistency was downgraded by 1. Funnel plots and statistical tests suggested the presence of publication bias, and publication bias was downgraded by 1. The 10 studies on lowering serum uric acid levels had high inter-study heterogeneity, but we found the source of heterogeneity and therefore did not downgrade. For the 2 studies on immediate analgesic effect, inconsistency and publication bias were each downgraded by 1 grade because of high heterogeneity and a small number of RCTs. Regarding adverse events, the number of RCTs was small and publication bias was downgraded by 1 grade. Overall, the level of evidence for lowering serum uric acid levels was intermediate, the level of evidence for effectiveness and incidence of adverse events was low, and the level of evidence for analgesic effect and immediate analgesic effect was very low. Detailed results are shown in Table 3. |  |
| **DISCUSSION** | | |  |
| Discussion | 23a | In our study, the heterogeneity of the ten studies on AGA pain was high. (P < 0.00001, I^2^ = 83%), and we performed a subgroup analysis, which showed that there was no significant heterogeneity among the four studies on the nonpharmacological electroacupuncture group and the pharmacological group alone (P = 0.76, I^2^ = 0%). Then, in six studies comparing the efficacy of electroacupuncture in combination with drugs versus conventional drugs, statistically significant heterogeneity was found. (P＜0.00001, I^2^=87%), for which we hypothesized that the source of heterogeneity might be related to different drug combinations. In addition, we found the source of heterogeneity in the study of serum uric acid levels by sensitivity analysis (P < 0.0001, I^2^ = 74%). When the study of Zou 2006 was excluded, there was less heterogeneity among the studies (P = 0.29, I^2^ = 17%). We believe this may be due to the fact that the study used electroacupuncture alone. | 16 |
|  | 23b | First, the completion of the RCTs in this study took place solely in China, without any multicenter trials being carried out. This could potentially impact the overall quality of evidence presented in this study.  Second, electroacupuncture, being an exceptional therapy within complementary alternative medicine, presents difficulty in establishing precise guidelines regarding the intensity of stimulation and the appropriate selection of sites for needle manipulation. Consequently, the assessment of electroacupuncture's effectiveness may be influenced by bias.  Third, according to the ACR guidelines, medication selection should be determined by the number of joints affected and the severity of pain. It is recommended that identified pharmacologic therapies for reducing uric acid levels should be continued without any breaks. Nevertheless, certain studies that were included in our analysis only provided first-line treatment options such as NSAIDs, corticosteroids, and colchicine, making no mention of the management of ULT. This omission may potentially influence the interpretation of the findings.  Fourth, there may be publication bias regarding trial design and outcome evaluation. We note that the outcome evaluations for both efficiency and analgesia used manually evaluated scales, and the possibility exists that this ultimately led to publication bias. | 17 |
|  | 23d | In summary, the mechanism of electroacupuncture for AGA is not fully understood and further studies are needed to clarify it. Interpretations of the results should be approached with caution due to the poor quality of the studies included. Studying future studies encourages additional randomized controlled trials with more rigorous designs and larger sample sizes. | 17 |
| **OTHER INFORMATION** | | |  |
| Registration and protocol | 24a | We are pre-registered with the review protocol (https:// www.crd.york.ac.uk/prospero/), registration number CRD42023450037. | 3 |
|  | 24c | There are no systematic reviews or meta-analyses for electroacupuncture in the treatment of acute gouty arthritis, so we adapted the intervention |  |
| Support | 25 | This study was supported by Sichuan Cadre Health Care Project (No. ZH2020-1901) & Sichuan Famous Chinese Medicine Studio Project | 17 |
| Competing interests | 26 | Authors declare no conflict of interest. | 17 |
| Availability of data, code and other materials | 27 | Data for the study can be obtained through a reasonable process from the authors, who can be reached at 313474951@qq.com |  |

*From:*  Page MJ, McKenzie JE, Bossuyt PM, Boutron I, Hoffmann TC, Mulrow CD, et al. The PRISMA 2020 statement: an updated guideline for reporting systematic reviews. BMJ 2021;372:n71. doi: 10.1136/bmj.n71

For more information, visit: <http://www.prisma-statement.org/>
