# Supplementary material for: Electroacupuncture for acute gouty arthritis: a systematic review and meta-analysis of randomized controlled trials
Source: Front Immunol. 2024 Jan 4;14:1295154. doi: 10.3389/fimmu.2023.1295154 (PMC10794621; doi:10.3389/fimmu.2023.1295154)

**Supplementary Appendix**

**Search strategies used for different databases**

**Boolean logic operators**

(((Gouty Arthritis [MeSH Terms] OR Gouty Arthritis [All Fields]) AND (Electroacupuncture [MeSH Terms] OR Electroacupuncture [All Fields])) AND (RCTs [MeSH] OR RCTs [All Fields]))

**Pubmed**

#1 ((((Electroacupuncture) OR (Electroneedle)) OR (Automated Acupuncture)) OR (Electroacupuncture Treatment)) OR (Treatment, Tlectroacupuncture)

#2 (Arthritis) OR (Acute Arthritis)

#3 (Gout) OR (Gouty)

#4 (((((Randomised controlled trial) OR (Controlled clinical trial)) OR (Randomised)) OR (Randomly)) OR (Trial)) OR (Groups)

#5 #1 AND #2 AND #3 AND #4


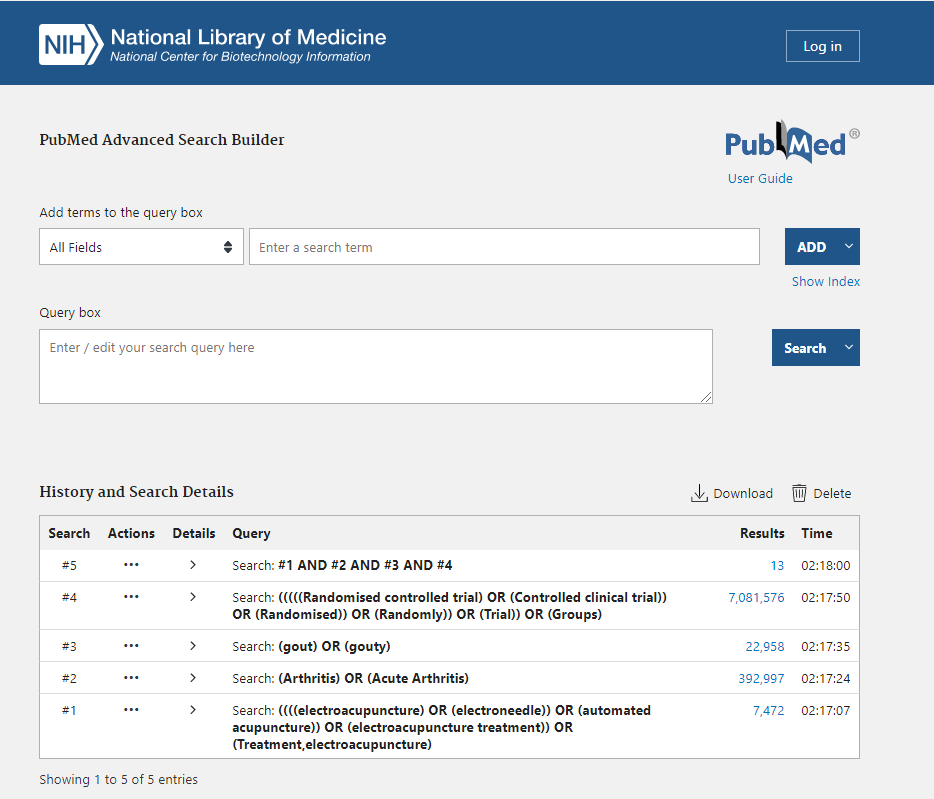


**Web of Science**

#1 ((((TS=(Electroacupuncture*)) OR TS=(Electroneedle*)) OR TS=(Automated Acupuncture*)) OR TS=(Electroacupuncture Treatment*)) OR TS=(Treatment,electroacupuncture*)

#2 (TS=(Arthritis*)) OR TS=(Acute Arthritis*)

#3 (TS=(Gout*)) OR TS=(Gouty*)

#4 (TS=((((((Randomised controlled trial) OR (Controlled clinical trial)) OR (Randomised)) OR (Randomly)) OR (Trial)) OR (Groups)))

#5 #1 AND #2 AND #3 AND #4


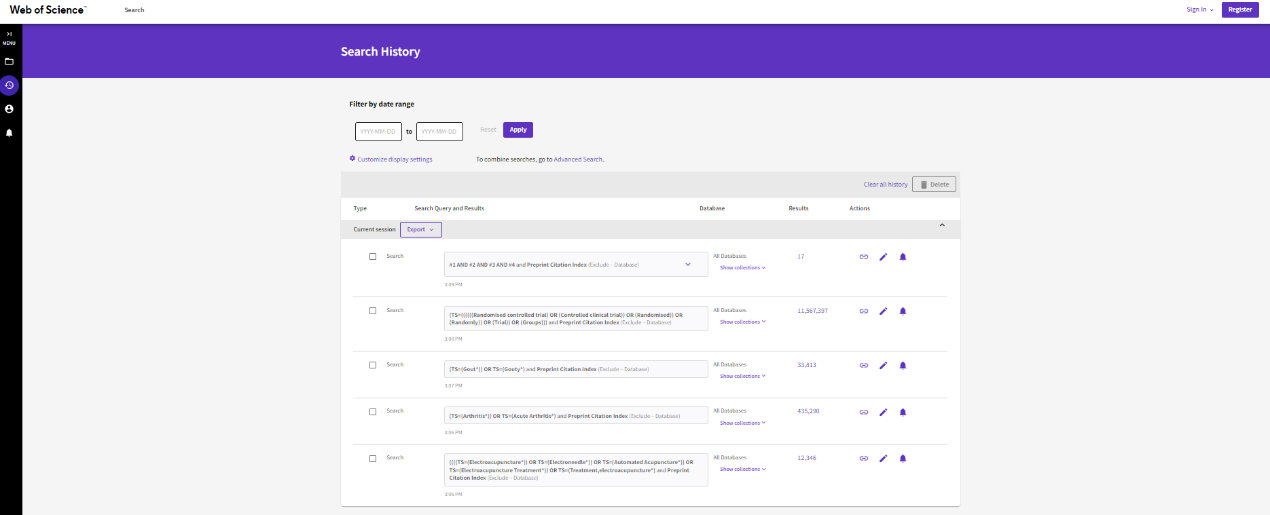


**Cochrane library**

#1 MeSH descriptor: [Electroacupuncture] explode all trees

#2 MeSH descriptor: [Arthritis] explode all trees

#3 MeSH descriptor: [Randomized Controlled Trial] explode all trees

#4 MeSH descriptor: [Randomized Controlled Trials as Topic] explode all trees

#5 MeSH descriptor: [Control Groups] explode all trees

#6 #2 OR #3 OR #4 OR #5 in Cochrane Reviews, Cochrane Protocols, Trials, Clinical Answers, Editorials, Special Collections

#7 MeSH descriptor: [Gout] explode all trees

#18 #1 AND #6 AND #7


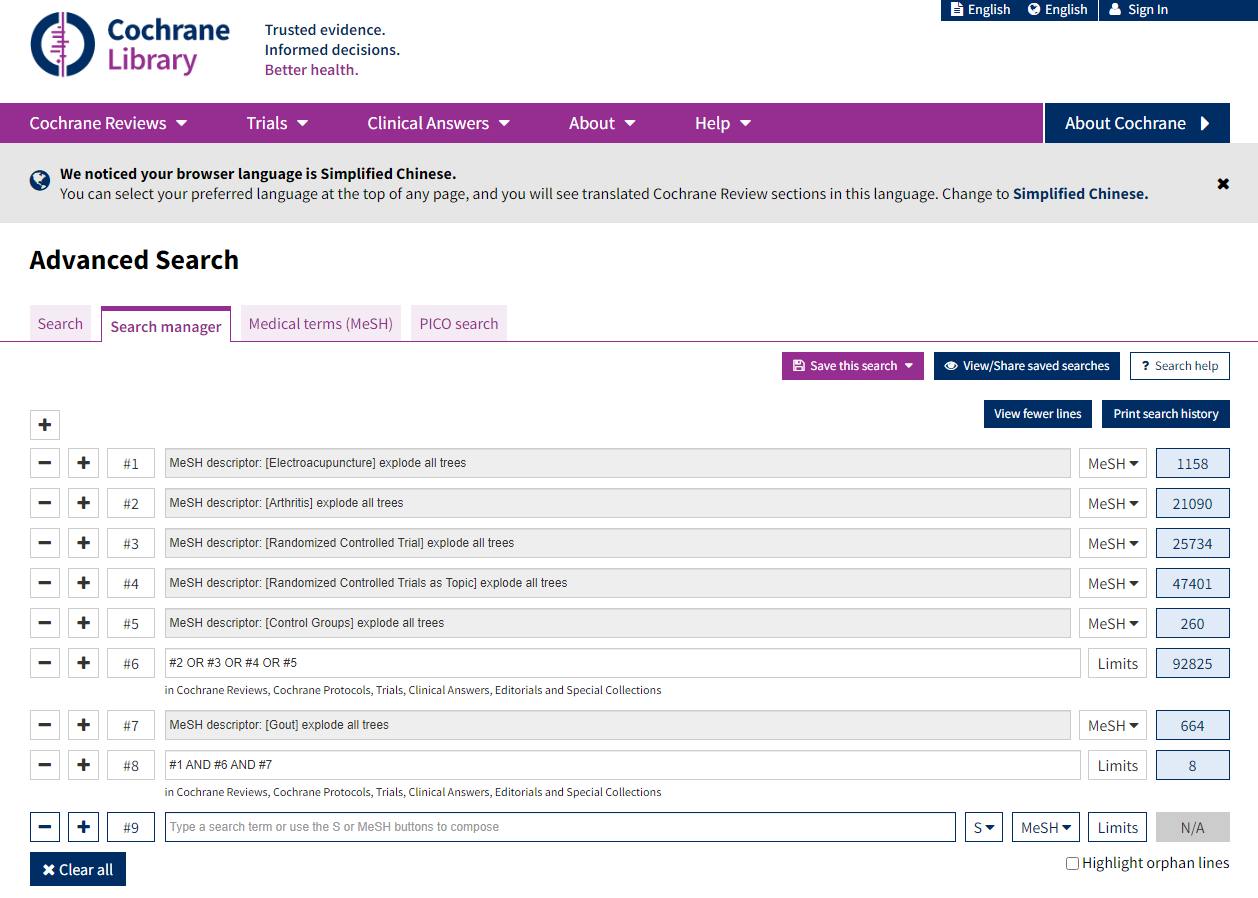


**Embase**

#1 ('electroacupuncture'/exp OR 'electroacupuncture treatment' OR 'electroacupuncture therapy')

#2 ('arthritis'/exp OR 'acute arthritis'/exp)

#3 ('gout'/exp)

#4(randomised AND controlled AND trial OR controlled)AND clinical AND trial OR randomised OR randomly OR trial OR groups)

#5 #1 AND #2 AND #3 AND #4


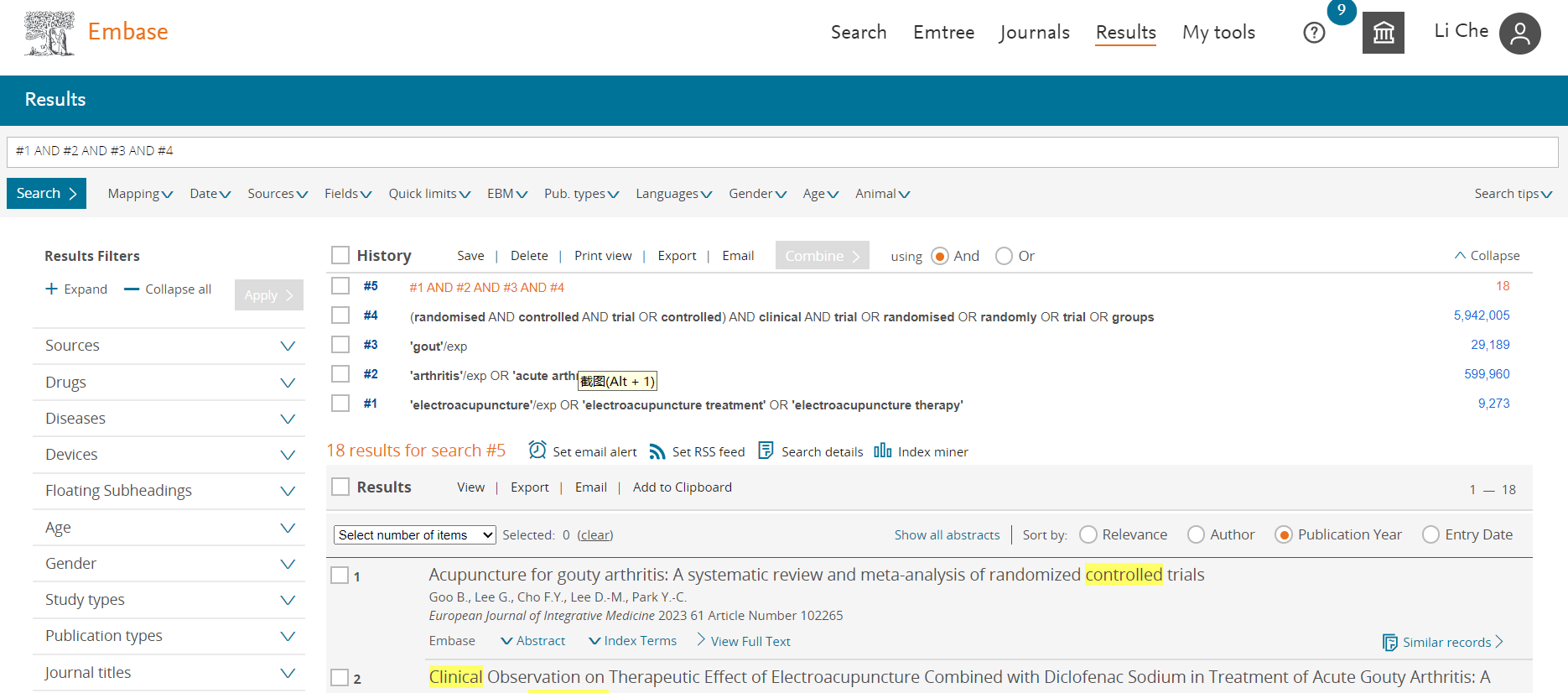


**CNKI**

(((SU = '急性关节炎'+'关节炎') OR (TKA = '急性关节炎'+'关节炎')) AND ((TKA = '电针') AND ((SU = 'RCT') OR (TKA = '随机' + '对照' + '分配' + '临床' + '试验' + '多中心' + '随访' + 'RCT' + 'CCT'))) AND ((SU%= '痛风') OR (TKA = '痛风')) NOT ((SU%= '动物实验') OR (TKA = '鼠') OR (TKA = '兔'))


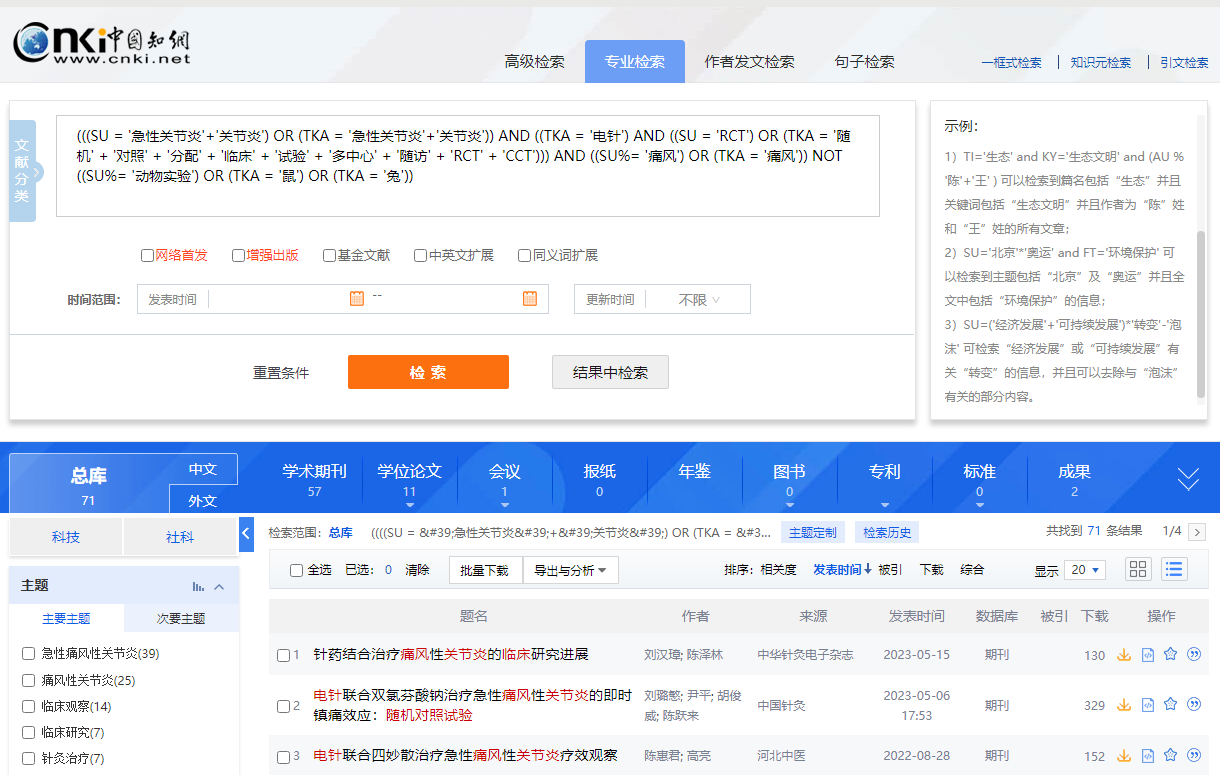


**CBM**

((电针 OR 电针治疗) AND (关节炎 OR 急性关节炎)) AND (痛风) AND (随机 OR 对照 OR 试验 OR 分组) NOT (鼠 OR 兔)


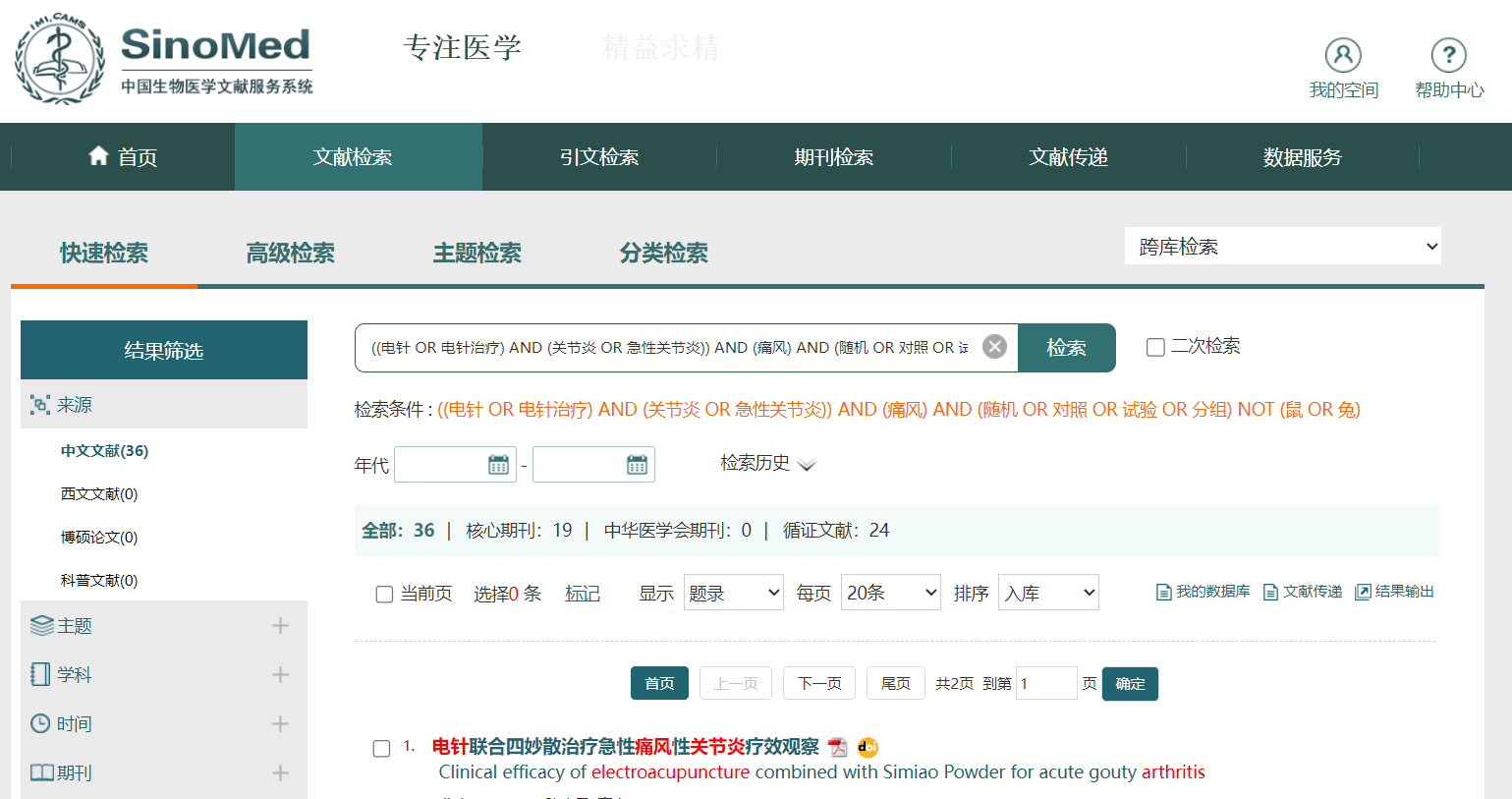


**VIP**

U=(电针 OR 电针治疗) AND U= (关节炎 OR 急性关节炎) AND U= (痛风) AND U= (随机 OR 对照 OR 试验 OR 分组)


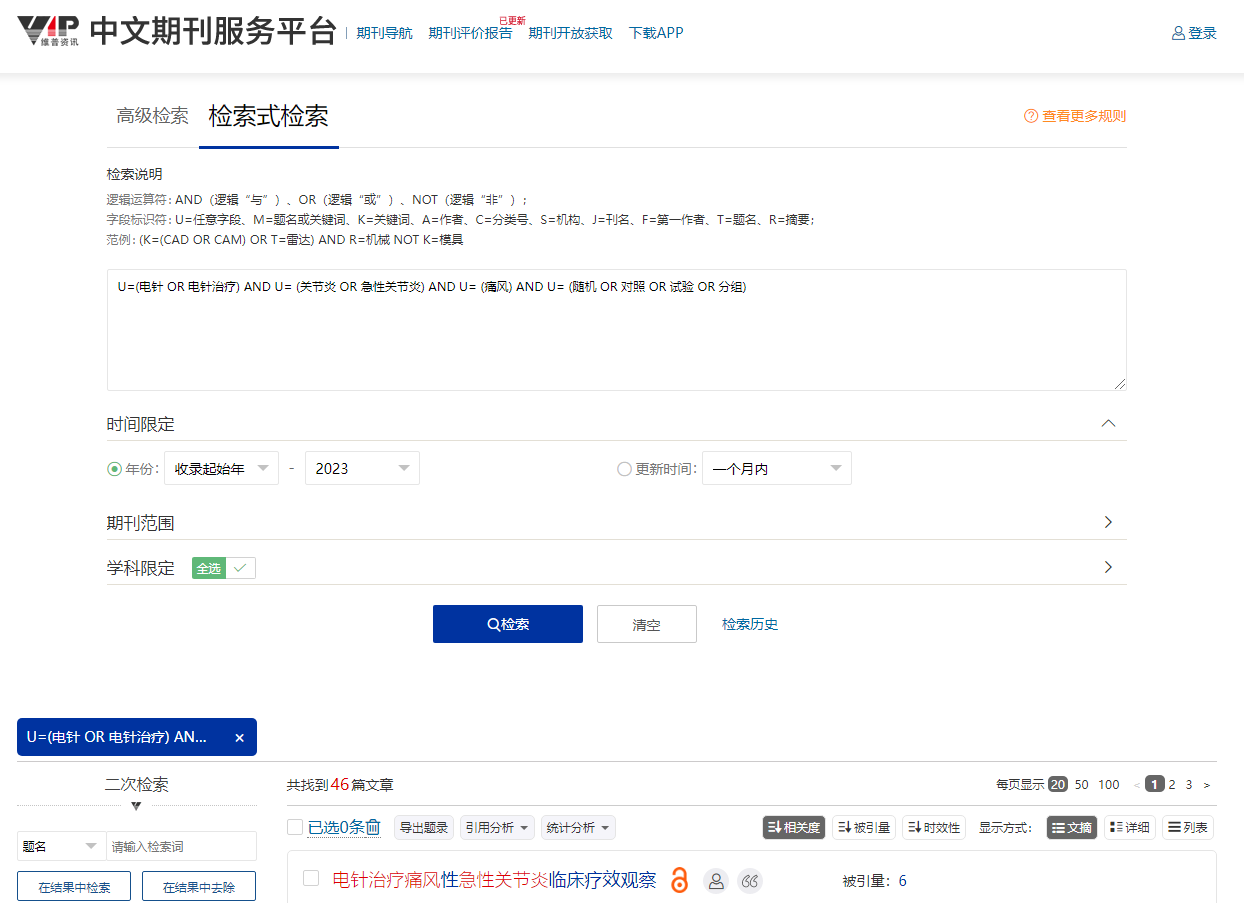


**Wan-fang**

主题=((电针 OR 电针治疗) AND (关节炎 OR 急性关节炎)) AND (痛风) AND (随机 OR 对照 OR 试验 OR 分组) NOT (鼠 OR 兔)


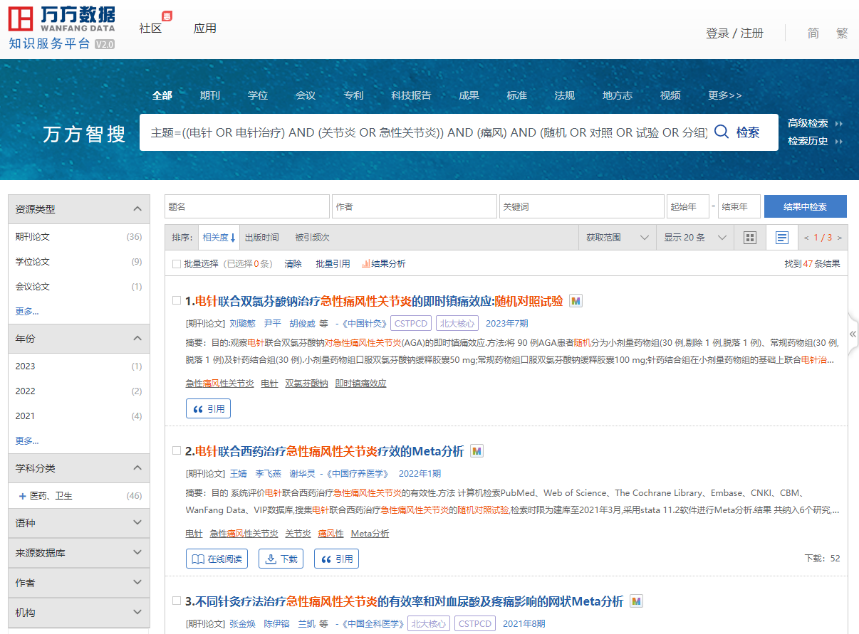

Supplement: Supplementary file 2 [file DataSheet_2.docx]
